# Molecular impacts of Meier–Gorlin syndrome mutations on human origin licensing

**DOI:** 10.1016/j.jbc.2025.111100

**Published:** 2025-12-23

**Authors:** Ran Yang, Olivia Hunker, Jenna Kim, Franziska Bleichert

**Affiliations:** Department of Molecular Biophysics and Biochemistry, Yale University, New Haven, Connecticut, USA

**Keywords:** DNA replication initiation, origin licensing, Meier-Gorlin syndrome, MCM loading, *in vitro* reconstitution

## Abstract

Meier–Gorlin syndrome (MGS) is a form of primordial dwarfism linked to mutations in DNA replication initiation factors. Many MGS variants affect proteins required for the first step of replication initiation—the licensing of replication origins—during which the origin recognition complex (ORC), CDC6, and CDT1 cooperatively load MCM2-7 complexes onto DNA as an MCM double hexamer. The specific impacts of MGS mutations on origin licensing remain poorly understood. In this study, we systematically analyze the effects of MGS-linked missense mutations in core domains of human origin licensing factors in a fully reconstituted *in vitro* MCM loading system. Our results show that MGS mutations inhibit origin licensing by blocking MCM recruitment or loading at discrete but distinct stages of the reaction. MGS mutations in ORC and CDC6 impair MCM recruitment by abrogating ATP-dependent DNA binding or the maturation of recruited MCM into a loaded single hexamer. MGS variants of CDT1 specifically reduce MCM recruitment, whereas disease mutations in MCM subunits support ORC-mediated MCM hexamer recruitment but hinder their stable deposition onto DNA. Our findings establish how MGS mutations perturb specific origin licensing steps and provide mechanistic insights into the molecular basis of MGS pathogenesis.

Successful replication of genomic DNA relies on the coordinated assembly of DNA replication machineries at eukaryotic replication origins in a two-step initiation reaction. First, two copies of the heterohexameric MCM2-7 complex, the helicase motor, are loaded onto DNA as a head-to-head double hexamer (MCM-DH) (1, 2, 3). This event—origin licensing—requires the concerted action of the origin recognition complex (ORC), the co-loader CDC6, and the licensing factor CDT1 for recruitment and loading of MCM (4, 5). When origins fire, MCM double hexamers are activated to form two opposing CMG helicases for bidirectional DNA replication (5, 6). Replication initiation is under stringent spatiotemporal regulation during the cell cycle, cell differentiation, and development to preserve genome integrity and ensure that DNA replication and cell division align with the demands of organismal growth (7, 8, 9, 10). Consequently, dysregulation of this process is linked to various human diseases (11, 12, 13).

Meier–Gorlin syndrome (MGS) is a rare genetic disease that has been linked to mutations in replication initiation factors, particularly those involved in origin licensing, including components of ORC, CDC6, CDT1, and MCM proteins (14, 15, 16, 17, 18). As a form of primordial dwarfism, MGS is associated with short stature, but other skeletal abnormalities (*e.g.*, microtia, absent or hypoplastic patella, microcephaly) are also observed in some patients (19). Given that pathogenic variants occur in genes encoding replication initiation factors, it is logical to assume that MGS phenotypes arise from defects in DNA replication (13). However, the molecular mechanisms underlying MGS pathogenesis remain poorly defined. Several initiation factors perform nonreplicative functions that, when perturbed, may contribute to MGS pathology (20, 21, 22, 23, 24, 25, 26). Notably, patient-derived cells can proliferate efficiently in culture, and poor correlations between replicative capacity of MGS cells and clinical phenotypes have been reported (26). Most importantly, it remains unclear whether all MGS-associated missense mutations—particularly those in origin licensing factors—impair replication initiation.

During origin licensing, ORC is the first initiation factor to bind origin DNA (27). ORC is then joined by CDC6 to form a ring-shaped complex on DNA, providing a platform for the recruitment of CDT1 and MCM into an ORC-CDC6-CDT1-MCM (OCCM) intermediate (28, 29). The OCCM loads an MCM hexamer onto DNA through an opening in the MCM ring between the MCM2 and MCM5 subunits (30); subsequently, this gate closes to retain MCM single hexamers (MCM-SH) on DNA (31, 32, 33). MCM double hexamers assemble by the dimerization of two DNA-loaded MCM rings through interlocking of their N-terminal domains (1, 2, 34). In the human system, the second MCM hexamer can be loaded through multiple discrete mechanisms that either involve ORC6 or occur independently of this ORC subunit (32, 33, 35). We previously showed that two MGS-associated missense mutations in ORC6 reduce MCM-DH formation by impairing the assembly of an MCM-ORC (MO) intermediate that likely orients ORC for the second MCM loading event (32). It has been hypothesized that MGS variants of other MCM loading factors also impede efficient origin licensing, but the extent to which these mutations impact MCM-DH formation and the mechanism by which they do so have yet to be uncovered.

Here, we set out to define whether and how origin licensing is more broadly deregulated in MGS by leveraging an *in vitro* reconstituted human MCM loading system we recently established (32). Through systematic biochemical analyses of MGS disease mutations in MCM loading factors, we demonstrate defects in origin licensing for all but one of the variants. We find that MGS mutations halt MCM loading at distinct stages of the licensing reaction through several mechanisms: impairing ORC binding to DNA, hindering ORC’s ability to sample an active conformation, altering ORC’s ATPase activity, interfering with MCM recruitment and OCCM assembly, or preventing MCM-DH formation following recruitment. Collectively, these findings rationalize how MGS-associated mutations compromise replication origin licensing, establish a direct mechanistic link between defects in DNA replication initiation and MGS, and support a model in which impaired DNA replication underlies MGS pathogenesis.

## Results

### MGS mutations in core ORC subunits impede origin licensing

Several ORC subunits have been found to harbor missense mutations in MGS patients, including ORC1, ORC4, and ORC6 (14, 15, 16). Within ORC, the ORC1-5 subunits contain an ATPases Associated with various cellular Activities (AAA+) fold and co-assemble into a pentameric ring (36, 37, 38); by contrast, ORC6 is structurally unrelated and associates peripherally with the complex, exhibiting only weak affinity for the other ORC subunits in the human complex (22, 38, 39, 40). While MGS substitutions in ORC6 inhibit formation of the MO intermediate after MCM-SH loading (32), we hypothesized that the mutations in other ORC subunits may perturb licensing through different mechanisms. These additional mutations cluster in two functionally distinct regions of ORC: the ATPase domains of ORC1 and ORC4, and the N-terminal bromo-adjacent homology (BAH) domain of ORC1 (14, 16, 19). As the ORC1 BAH domain does not directly contribute to MCM loading in the reconstituted *in vitro* system (32), we focused our analysis on mutations in the AAA+ domains.

**Figure 1:**
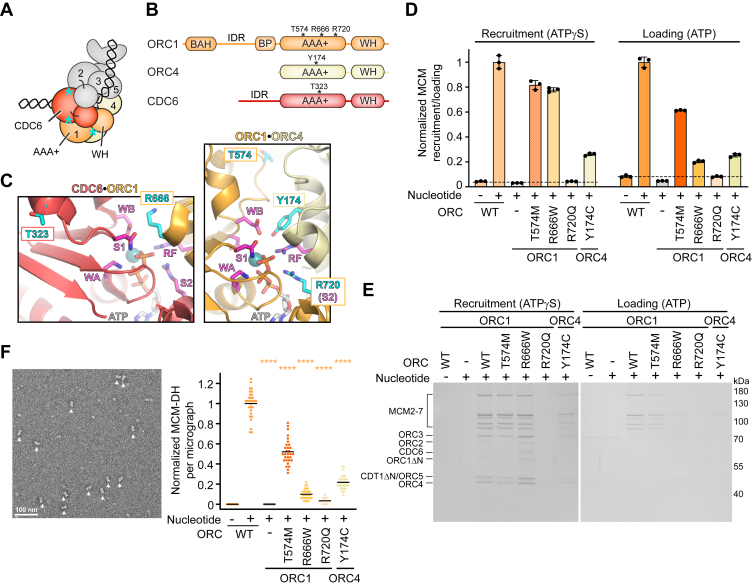
**Meier–Gorlin syndrome mutations in human ORC have different impacts on MCM recruitment *in vitro* and impede MCM loading through distinct mechanisms.***A*, schematic of the ORC–DNA–CDC6 loading intermediate poised for MCM recruitment. The location of MGS mutations in ORC and CDC6 are approximated by *cyan asterisks*. *B*, domain architecture of ORC1, ORC4, and CDC6. MGS mutations occur in the AAA+ domains that form the ORC1•ORC4 and CDC6•ORC1 ATPase centers in the ORC–DNA–CDC6 complex and near the CDC6•ORC2 interface. *C*, zoomed views of the CDC6•ORC1 and the ORC1•ORC4 ATPase sites of the *Drosophila* ORC–DNA–CDC6 complex (PDB 7JK4 (28), the structure of the human equivalent has not yet been determined) highlighting the location of MGS mutations (in *cyan*) and active site residues important for ATP binding and hydrolysis (in *magenta*). Note that arginine 666 in human ORC1 is a lysine in *Drosophila* ORC1. Mg^2+^ is depicted as *teal sphere*. *D*, MCM-GFP fluorescence measurements in the elutions of bead-based MCM recruitment and loading reactions with WT ORC, ORC containing ORC1 and ORC4 MGS variants, and an ORC2-5 complex (−). MCM-GFP fluorescence was normalized to the average GFP signal of reactions done with WT ORC and nucleotide. The means and SDs of three independent experiments are plotted. *Dashed* lines mark background fluorescence in reactions without nucleotide. *E*, silver-stained SDS-PAGE gel of elutions from recruitment and loading reactions done with WT or MGS-mutant ORC. *F*, quantification of MCM double hexamer (MCM-DH) particles in the elutions of MCM loading reactions. A representative electron micrograph is shown on the *left* for a reaction with WT ORC, with MCM-DH particles marked by *arrowheads*. MCM-DH particles were counted on ten electron micrographs per repeat (total *n* = 30) for each condition and normalized to reactions done with WT ORC. *Black* bars in scatter plot represent means. Statistical significance (compared to WT ORC) was calculated with the two-way ANOVA and Tukey’s multiple comparison test. ∗∗∗∗*p* < 0.0001. All loading assays throughout the manuscript unless noted otherwise were done with N-terminally truncated ORC1 and CDT1 (ORC1ΔN and CDT1ΔN), which support MCM loading with the same efficiency as full-length proteins (32). MGS, Meier–Gorlin syndrome; ORC, origin recognition complex; BAH, bromo-adjacent homology domain; BP, basic patch; WH, winged helix domain; WA, Walker A; WB, Walker B; S1, sensor 1; S2, sensor 2; RF, arginine finger.

ORC1 and ORC4 contribute to two functional ATPase sites. One of these is located at the ORC1•ORC4 interface and the other at the CDC6•ORC1 interface, which forms when CDC6, itself an AAA+ ATPase, joins the ORC ring (28, 41, 42, 43) (Fig. 1*A*). Mapping MGS mutations in ORC1 and ORC4 onto the cryo-EM structure of a DNA-bound *Drosophila* ORC–CDC6 complex ((28), an equivalent structure of an isolated human ORC–DNA–CDC6 complex has not yet been determined) revealed that the affected residues either directly participate in ATPase site formation (ORC1^R666^, ORC1^R720^, ORC4^Y174^) or lie in close proximity (ORC1^T574^), suggesting they may interfere with the function of these sites (Fig. 1, *A*–*C*). Indeed, two of the MGS variants, ORC1^R720Q^ and ORC4^Y174C^, have been reported to reduce ATPase activity at the ORC1•ORC4 interface (44). The MGS residue in CDC6 (T323) likewise resides near an ATPase site (Fig. 1, *A*–*C*). However, ATP hydrolysis by both ORC and Cdc6 are not required for MCM loading *in vitro* in yeast (45, 46), and human ORC’s ATPase activity is also dispensable in this reaction (35). These findings raise questions about the functional consequences of these MGS mutations for origin licensing.

To address directly whether MGS variants in ORC subunits impede origin licensing, we purified MGS-mutant human ORC1-5 assemblies (referred to as ORC hereafter), as well as an ORC2-5 complex lacking ORC1, and tested their abilities to support human MCM recruitment and loading using our recently established *in vitro* reconstitution system (32) (Fig. S1*A*). WT or MGS-mutant ORC1-5 were incubated with ORC6 (purified separately), other loading factors, and bead-coupled DNA in ATP or ATPγS conditions and then subjected to low- or high-salt washes to assess MCM recruitment and loading, respectively. We used GFP-labeled MCM (GFP on MCM2 (32)) so that the amount of MCM retained on DNA after washes could be quantified by fluorescence measurement upon elution by nuclease digestion, in parallel with detecting proteins by SDS-PAGE and silver staining. Using this setup, we found that all MGS variants apart from ORC1^T574M^ caused a strong MCM-loading defect (Fig. 1, *D* and *E*), which we confirmed by counting MCM double hexamers in the elutions of loading reactions by negative-stain electron microscopy (EM) as done previously (32) (Fig. 1*F*). MCM loading was impaired the least by ORC1^T574M^ (Fig. 1, *D*–*F*); this defect with ORC1^T574M^ could be accounted for by destabilization of the initiator complex that was prevalent in multiple ORC preparations and increased over time, compelling us to exclude this mutant from further analysis (Fig. S1*B*). Notably, the decrease in MCM double hexamer formation with the other ORC MGS variants was more striking and did not strictly correlate with defects in MCM recruitment; while MCM loading was impaired to a similar extent with ORC1^R666W^ and ORC4^Y174C^, ORC1^R666W^ but not ORC4^Y174C^ supported MCM recruitment to almost WT levels and increased the retention of ORC, CDC6, and likely also CDT1 on DNA in ATPγS conditions (Fig. 1, *D*–*F*). These disparate effects of ORC MGS variants on MCM recruitment *versus* loading suggest that the mutations impede origin licensing by distinct mechanisms.

### MGS mutations in ORC inhibit initiator binding to DNA and alter ORC’s basal ATPase rate

**Figure 2:**
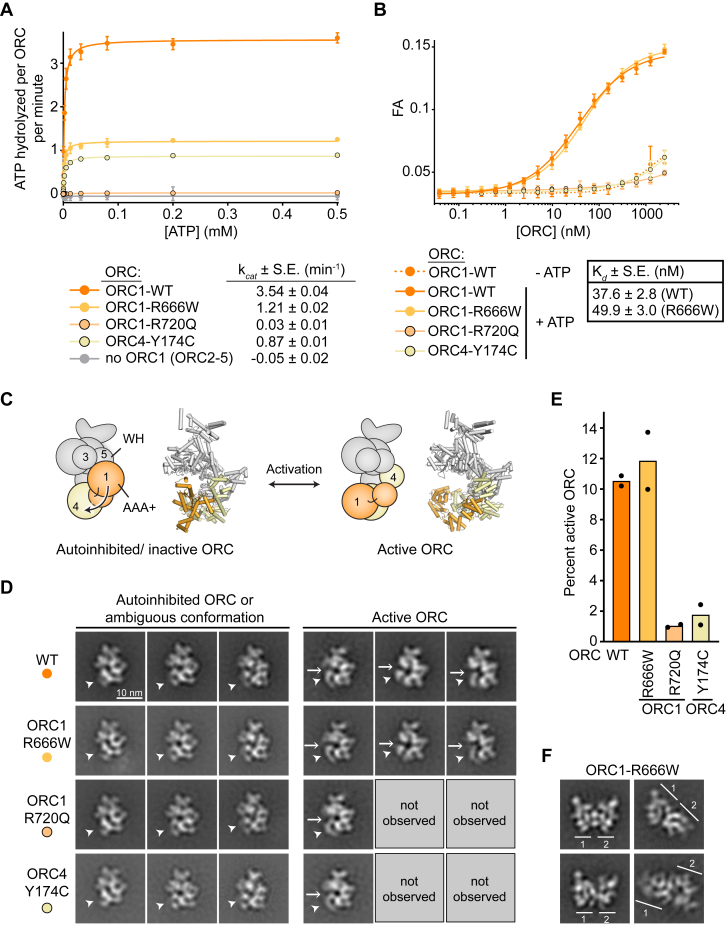
**MGS mutations in ORC1 and ORC4 alter distinct ORC activities.***A*, all MGS mutations in ORC reduce ORC’s ATPase rate. Michaelis–Menten curves for ATP hydrolysis rate measurements from three independent experiments (mean and SD) are shown. k_*cat*_ values (means and standard errors) are listed. *B*, MGS mutations at the ORC1•ORC4 ATPase site (ORC1^R720Q^ and ORC4^Y174C^) abrogate ATP-dependent, high-affinity DNA binding by ORC. Fluorescence anisotropy measurements (means and SDs from three independent experiments) of human ORC binding to fluorescently labeled DNA are plotted. Dissociation constants (K_*d*_) and standard errors (S.E.) are listed. *C*–*E*, ORC-MGS mutations ORC1^R720Q^ and ORC4^Y174C^ destabilize the active ORC conformation. *C*, schematic of autoinhibited/inactive and activated ORC states. *D*, negative-stain EM 2D class averages of WT and MGS-mutant ORC assemblies. *Arrowheads* point towards ORC1 in class averages. *Arrows* mark the gap in the ORC ring in the active state that provides access to ORC’s central channel. *E*, quantification of ORC particles in the active conformation for WT and MGS-mutant ORC assemblies from two independent experiments each. We note that the percentage of active ORC (particularly with WT ORC) is likely an underestimation because the two conformations cannot be unambiguously distinguished in all views of the complex; these ambiguous views were not counted as active state particles. *F*, ORC with ORC1^R666W^ has a propensity to dimerize. Negative-stain 2D EM class averages of dimers are shown. Scale is the same as in *panel* (*D*).

To delineate how MGS mutations in ORC1 and ORC4 hamper ORC function during origin licensing, we examined their impacts on two ORC activities, ATP hydrolysis and ATP-dependent DNA binding. We first measured the steady-state ATPase activity of human ORC assemblies using an NADH-coupled assay with ATP regeneration, which reports on ATP hydrolysis at the ORC1•ORC4 site (28). These experiments yielded an ATPase rate of 3.5 ATPs hydrolyzed per minute for each wildtype ORC, slightly lower than the rate measured previously with the *Drosophila* complex (∼9 ATPs per ORC per minute) (28), while an ORC2-5 complex lacking ORC1 did not hydrolyze ATP as expected (Fig. 2*A*). All MGS-mutant ORC assemblies were compromised in hydrolyzing ATP, with the largest ATP hydrolysis defect observed with ORC1^R720Q^ (Fig. 2*A*). This ORC1^R720^ constitutes the sensor 2 residue, a key element in the ORC1•ORC4 active site which directly interacts with ATP; substitution of this arginine to glutamine is expected to compromise this activity (Fig. 1*C*). The Y174 residue in ORC4, on the other hand, hydrogen bonds with the Walker B residue, a motif critical for ATP hydrolysis; changing this tyrosine to cysteine likely alters the conformation of the Walker B motif and consequently ATPase activity. The ATP hydrolysis defect with ORC1^R666W^ was more surprising as R666 does not reside in the ORC1•ORC4 ATPase site; however, this mutation may alter the conformation of a nearby region harboring the ORC1 sensor 1, a residue critical for ATPase activity, and thereby impart an effect on the activity at the ORC1•ORC4 ATPase site.

ORC’s ATP-dependent DNA binding activity is mediated by its central channel through multiple DNA contact sites in the AAA+ and winged-helix domains of ORC subunits (28, 38, 47, 48). Using fluorescence anisotropy, we measured the affinity of wildtype or mutant ORC for binding a 40 bp duplex DNA substrate. As seen previously with *Drosophila* ORC, wildtype human ORC binds DNA with nanomolar affinity in an ATP-dependent manner (48) (Fig. 2*B*). Strikingly, ORC1^R720Q^ and ORC4^Y174C^ completely abrogated ORC’s ability to bind DNA in this assay, rationalizing both the MCM recruitment and loading defects observed with these MGS variants (Fig. 2*B*). By contrast, ORC containing ORC1^R666W^ bound DNA with comparable affinity to wildtype ORC, consistent with its ability to recruit MCM to DNA (Fig. 2*B*).

While the ATP hydrolysis defects observed upon mutating ORC1^R720^ and ORC4^Y174^ could be rationalized by an altered interaction network or configuration of catalytic residues at the ORC1•ORC4 ATPase sites, the loss of ATP-dependent DNA binding could not. However, prior studies have shown that metazoan ORC assemblies can adopt different conformational states: an active state, in which ORC1 and ORC4 AAA+ domain are juxtaposed, and an autoinhibited state, in which the ORC1•ORC4 interface is disrupted due to a reorientation of the ORC1 AAA+ domain and access to the DNA binding channel is sterically blocked (38, 48, 49) (Fig. 2*C*). We hypothesized that the two MGS mutations at the interface between ORC1 and ORC4 (ORC1^R720Q^ and ORC4^Y174C^) may destabilize the active state, thereby impeding DNA binding by ORC. To test this premise, we turned to negative-stain EM to directly visualize wildtype and mutant ORC assemblies. As seen in previous negative-stain EM of *Drosophila* and human ORC (48), 2D classification of wildtype ORC particles yielded class averages corresponding to both active ORC and autoinhibited ORC, with ∼11% of total ORC complexes being in the active state (this number is an underestimation since many views preclude the distinction of the two ORC1 conformations) (Fig. 2, *D* and *E*). Strikingly, the abundance of ORC particles that are clearly in the active conformation dropped 5 to 10-fold with ORC1^R720Q^ and ORC4^Y174C^ but not with ORC1^R666W^. Overall, the reduced ability to sample the active ORC state explains the DNA binding defects exhibited by MGS-mutant ORC assemblies with ORC1^R720Q^ or ORC4^Y174C^ since this activity relies on a stable ORC1•ORC4 interface. Moreover, disruption of ORC1•ORC4 interactions likely also contributes to the strong reduction in ATPase rates seen with both ORC mutants. Intriguingly, we repeatedly observed well-defined class averages of ORC dimers with ORC1^R666W^ (Fig. 2*F*); similar class averages were rarely seen with wildtype ORC or the other mutant assemblies. Mass photometry of this ORC mutant also indicated an increase in the abundance of ORC dimers (Fig. S1*C*). Currently, we do not understand the functional significance of these ORC dimers and if they interfere with MCM loading directly, but these possibilities are interesting avenues for future work.

### ORC1^R666W^ and mutations in CDC6 impact the OCCM stage of MCM loading

**Figure 3:**
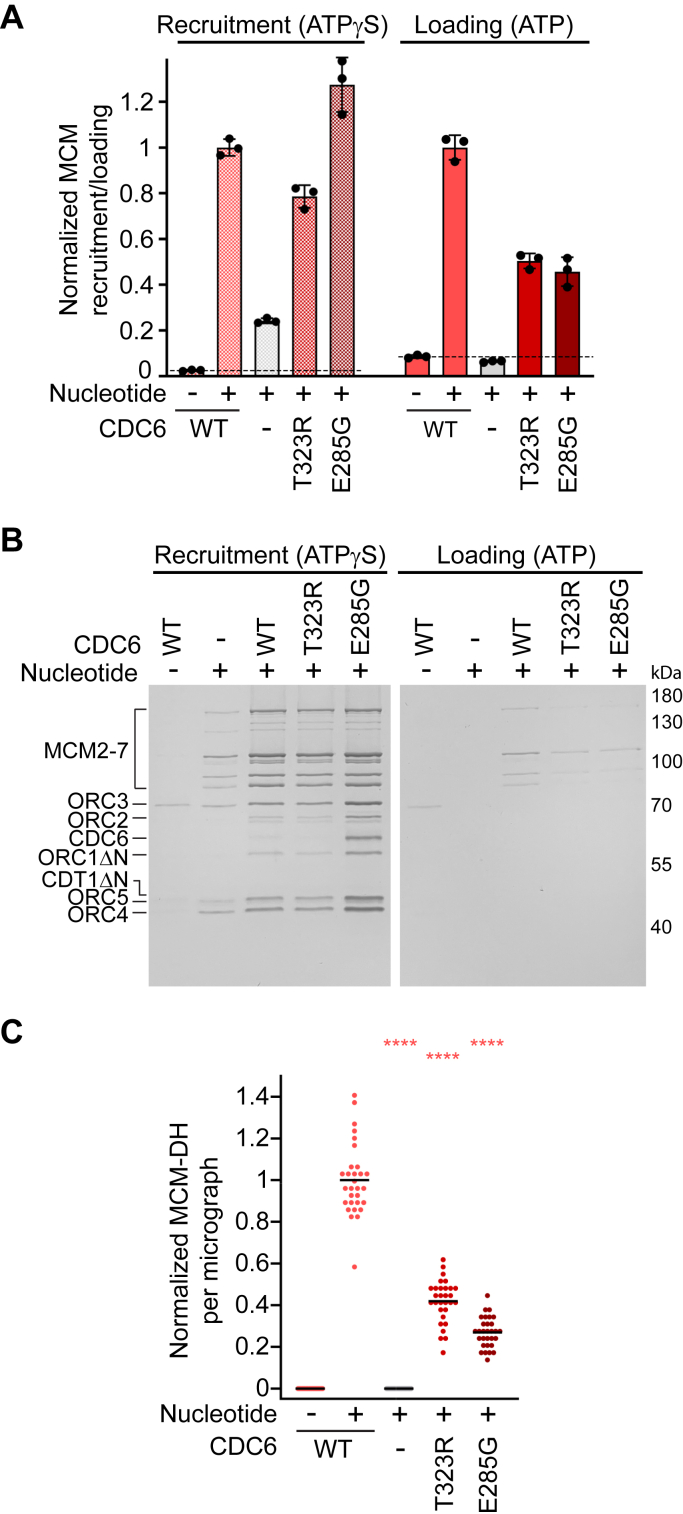
**CDC6 mutations near the CDC6•ORC1 ATPase site inhibit human origin licensing.***A*, normalized (to WT CDC6 reactions) MCM-GFP fluorescence in the elutions of MCM recruitment and loading reactions. Means and SDs of three independent experiments are plotted. *Dashed lines* mark background fluorescence in reactions without nucleotide. *B*, silver-stained SDS-PAGE gel of proteins eluted from DNA at the end of MCM recruitment and loading assays. *C*, MCM-DH particles per electron micrograph, normalized to MCM loading reactions with WT CDC6, that were eluted from DNA after high-salt wash. *n* = 30, with 10 micrographs analyzed per condition per three independent repeats. *Black* bars represent means. Statistical significance (compared to WT CDC6) was calculated with the two-way ANOVA and Tukey’s multiple comparison test. ∗∗∗∗*p* < 0.0001.

During origin licensing, CDC6 is recruited to DNA-bound ORC in an ATP-dependent manner, which establishes the second ATPase site (CDC6•ORC1) in the ORC-CDC6 complex (28, 43, 50, 51) (Fig. 1*A*). In budding yeast, ATP hydrolysis by Cdc6 contributes to a quality control pathway, as well as ORC-Cdc6 and OCCM disassembly (42, 45, 46, 52, 53). Of the ORC MGS variants, ORC1^R666W^ maps to the CDC6•ORC1 interface (Fig. 1, *A* and *C*). When analyzing this mutant in MCM recruitment reactions with ATPγS, we noticed an increase in the retention of ORC and CDC6 on DNA compared to reactions with wildtype ORC (Fig. 1*E*); this result could indicate that ORC1^R666W^ impairs ATP hydrolysis at this site in addition to the ORC1•ORC4 site (note that many ATPases can slowly hydrolyze ATPγS). Since we cannot directly measure the ATPase activity of human CDC6, we generated a catalytically inactive CDC6 mutant by changing the catalytic glutamate (E285) in the Walker B motif to glycine (an analogous mutation abrogates ATPase activity in the budding yeast ortholog (42, 43, 50)) and examined its effect in MCM recruitment and loading assays Fig. S1*D*). Like ORC1^R666W^, CDC6^E285G^ stabilized ORC and CDC6 on DNA during low-salt washes of MCM recruitment reactions but decreased MCM loading efficiency (Figs. 1, *D*–*F*, and 3, *A*–*C*).

We also tested the impact of MGS variant T323R in the AAA+ domain of CDC6 on origin licensing. CDC6^T323^ is located in a small pocket near the sensor 1 residue and the ORC2•CDC6 interface, which is likely distorted when the small, polar threonine side chain is replaced by a large, positively charged arginine (Fig. 1, *A* and *C*). In MCM recruitment assays, CDC6^T323R^ did not enhance retention of ORC, CDC6, and MCM on DNA as seen with CDC6^E285G^ but slightly impeded MCM recruitment (Figs. 1, *A* and *C*, 3, *A*–*C* and S1*D*). This mutant also led to a slightly weaker CDC6 signal in SDS-PAGE gels of MCM recruitment assays compared to wildtype CDC6 (Fig. 3*B*). In MCM loading assays, T323R reduced MCM double hexamer formation to ∼40 to 50% compared to wildtype CDC6 (Fig. 3, *A*–*C*).

Collectively, these results demonstrate that ORC1^R666W^ and CDC6 mutations in the AAA+ domain target the OCCM stage of the MCM loading reaction. These variants either impede CDC6 and MCM binding to ORC or hinder OCCM disassembly, potentially trapping unproductive loading intermediates and inhibiting MCM single hexamer loading onto DNA. These mechanisms differ fundamentally from those associated with ORC1^R720Q^ and ORC4^Y174C^, which interfere with origin licensing by abrogating DNA binding in ORC’s central channel – an essential step for subsequent CDC6 and MCM recruitment. However, the final outcome of all mutations is a reduction in MCM double hexamer assembly at origins.

### MGS mutations in CDT1 reduce the efficiency of origin licensing by impairing MCM recruitment

The ORC-CDC6 ring is a platform for recruiting the licensing factor CDT1 and MCM, both of which are also mutated in patients with MGS (14, 16, 17, 18). A prior study had shown that several CDT1 MGS variants, when expressed in U2OS cells, reduce CTD1 association with MCM and the amount of MCM on chromatin (54). However, it remains unclear if these mutations directly hinder MCM recruitment or loading. Therefore, we tested the effects of CDT1 missense mutations in our biochemical reconstitution system to define which step is affected.

**Figure 4:**
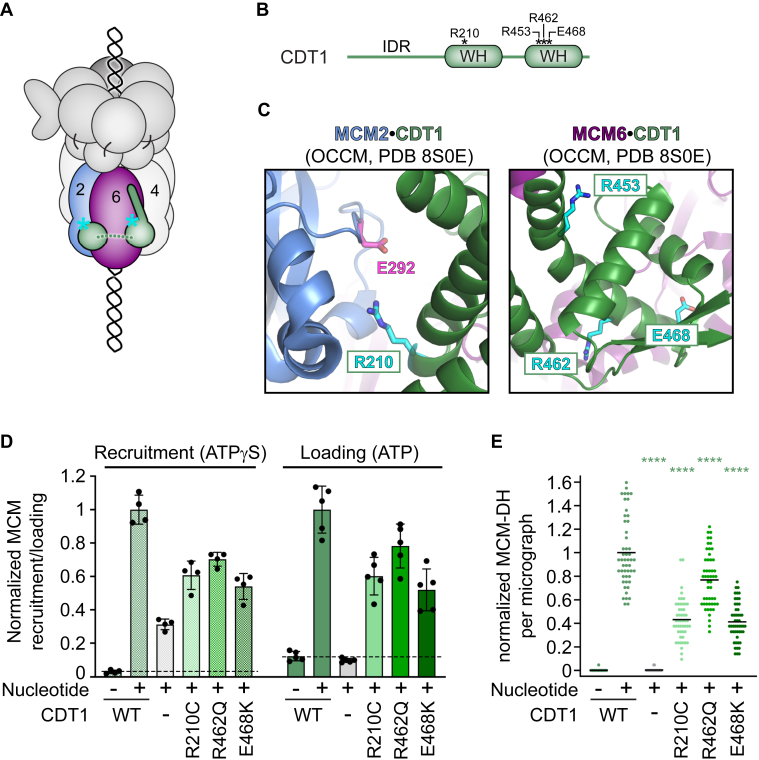
**MGS mutations in CDT1 diminish origin licensing by hindering MCM recruitment.***A*, schematic of the ORC-CDC6-CDT1-MCM (OCCM) intermediate. The location of MGS mutations is approximated by *cyan* asterisks. *B*, domain architecture of CDT1. Altered residues are mapped onto the respective domains. *C*, zoomed views of CDT1•MCM interfaces in the OCCM intermediate (PDB 8S0E (33)), with the mutated residues colored *cyan*. R210 in CDT1’s first WH domain is positioned near E292 of MCM2 (in *magenta*; *leftpanel*). MGS mutations in the second WH domain of CDT1 likely destabilize the protein fold or weaken interactions with MCM6 (*rightpanel*). *D* and *E*, CDT1 mutations reduce MCM recruitment and loading efficiency. *D*, MCM-GFP fluorescence in the elutions of MCM recruitment and loading assays, normalized to the average signal in reactions with WT CDT1. Means and SDs are plotted for *n* = 4 independent experiments for MCM recruitment and *n* = 5 independent experiments for MCM loading. *Dashed* lines mark background fluorescence in reactions without nucleotide. *E*, MCM-DH particle quantification per electron micrograph (after high-salt wash), normalized to the WT CDT1 sample. *n* = 50 micrographs per sample, with 10 images recorded per sample for each of the five repeat experiments. *Black* lines represent means. Statistical significance (compared to WT CDT1) was tested using two-way ANOVA analyses with Tukey’s multiple comparison test. ∗∗∗∗*p* < 0.0001. Full-length CDT1 was used in (*D* and *E*).

CDT1 is a key component of the OCCM licensing intermediate, interacting with MCM2, MCM4, and MCM6 in this complex (29, 33, 35, 55) (Fig. 4*A*). Human CDT1 bears two winged helix (WH) domains that are preceded by an N-terminal intrinsically disordered region (IDR; Fig. 4*B*). Several MGS mutations in CDT1 cluster in its second, C-terminal WH fold that interacts with MCM6’s N-terminal domain (NTD) (Fig. 4, *A*–*C*). Additional MGS variants have been identified in CDT1’s N-terminal IDR but we excluded these from our analyses as this region does not contribute to MCM recruitment and loading *in vitro* (32). Instead, we included the R210C hypomorphic variant in CDT1’s first WH domain that was discovered in flies (although not in MGS) and has been reported to reduce origin licensing in U2OS cells and *Xenopus* (54, 56, 57) (Fig. 4, *A*–*C*). We expressed and purified wildtype and mutant full-length CDT1 to test their activities in *in vitro* MCM recruitment and loading assays (Fig. S2*A*). Despite several attempts, we could not obtain sufficient amounts of CDT1^R453W^ as this protein was poorly expressed and prone to degradation. Inspection of OCCM-like structures showed that R453 is surrounded by other CDT1 residues, and changing this sidechain to tryptophan likely disrupts the WH fold and destabilizes the protein (Fig. 4*C*).

All purified CDT1 mutants (CDT1^R210C^, CDT1^R462Q^, and CDT1^E468K^) showed moderate disruption of both MCM recruitment and loading in *in vitro* origin licensing assays (Figs. 4, *D* and *E*, and S2*B*). As seen previously, a small amount of MCM could be recruited in the absence of CDT1 (32). Strikingly, the extent of MCM loading impairment caused by each mutant correlated closely with the decrease in MCM recruitment, suggesting that these mutations hinder MCM loading at the recruitment stage (Fig. 4, *D* and *E*). Since CDT1 is an essential component of the OCCM intermediate, we conclude that these amino acid substitutions likely destabilize CDT1-MCM interactions in the OCCM and perturb assembly of this intermediate. Indeed, the R210 residue is located at the MCM2-CDT1 interface and may form a salt bridge with E292 in MCM2, which would be abolished when this residue is replaced by cysteine (Fig. 4*C*). Likewise, R462 and E468 are located near the MCM6-CDT1 interface and likely contribute to the stability of MCM-CDT1 interactions (Fig. 4*C*). Our results are consistent with CDT1 being an integral component of the OCCM.

### All but one MGS variant in MCM subunits support MCM recruitment but hinder MCM loading

**Figure 5:**
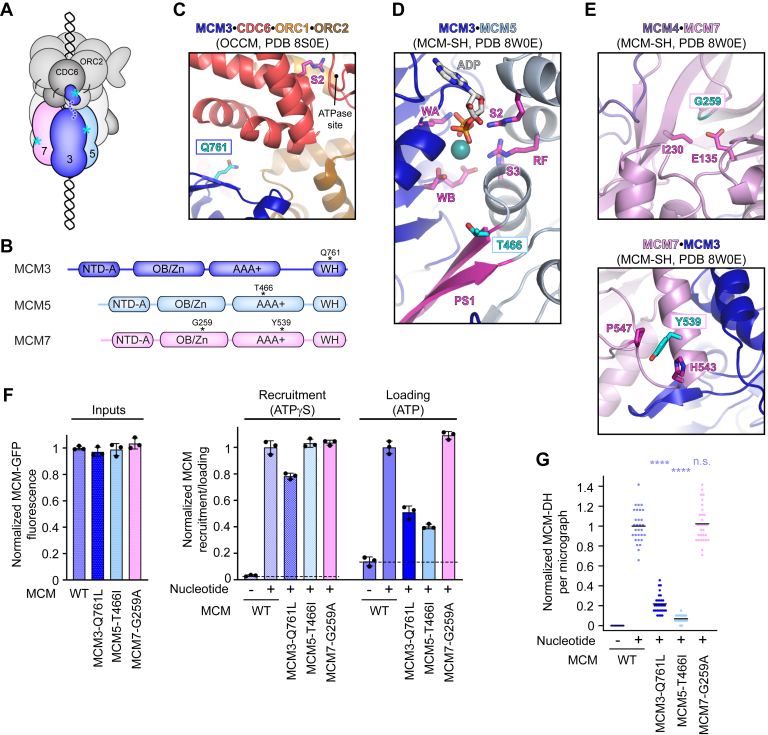
**MGS mutations in MCM subunits impede different steps in origin licensing.***A*, schematic of the OCCM with mutations in MCM subunits (in color) marked by *cyan* asterisks. *B*, domain architecture of MCM subunits mutated in MGS and altered residues indicated. *C*–*E*, MGS mutations in MCM subunits map to distinct sites in MCM loading intermediates. *C*, Q761 in MCM3 in the OCCM complex (PDB 8S0E (33)). *D*, T466 in MCM5 in the loaded MCM-SH structure (PDB 8W0E (32)). *E*, G259 (*top* panel) and Y539 (*bottom* panel) in the loaded MCM-SH structure (PDB 8W0E (32)). Residues mutated in MGS are colored *cyan*, while nearby amino acids or structural elements (*i.e.*, ATPase site residues) are colored *magenta*. *F* and *G*, MGS mutations in MCM have variable effects on MCM recruitment and loading. *F*, MCM-GFP fluorescence in inputs and elutions of MCM recruitment and loading assays, normalized to the average signal in reactions with WT MCM. Means and SDs for *n* = 3 independent repeat experiments are plotted. *Dashed* lines mark background fluorescence in reactions without nucleotide. *G*, MCM-DH particle quantification per electron micrograph (in the elutions of loading assays), normalized to the number of MCM-DHs seen in reactions with WT MCM. *n* = 30 micrographs per sample, with 10 images analyzed for each of the three repeat experiments. *Black* lines represent means. Statistical significance (compared to WT MCM) was calculated by two-way ANOVA analyses with Tukey’s multiple comparison test. ∗∗∗∗*p* < 0.0001, n.s. – not significant. NTD-A, N-terminal domain A; OB/Zn, oligonucleotide/oligosaccharide-binding fold/zinc finger.

Multiple MCM subunits have been reported to harbor missense mutations in MGS patients, including MCM3, MCM5, and MCM7 (17, 18) (Fig. 5, *A* and *B*). Unlike other licensing factors (*i.e.*, ORC, CDC6, and CDT1), the MCM complex is an integral component of replisomes and plays a central role in both initiation steps, origin licensing and origin firing. Yet, which initiation step is specifically impaired by MGS mutations in MCM remains unknown.

Therefore, we set out to examine the outcome of MGS missense mutations in MCM subunits on origin licensing. MCM3^Q761L^ maps to the WH domain at the C-terminus of the protein (Fig. 5, *A* and *B*). In the OCCM, this domain packs against CDC6 and ORC2 and creates an early tether for MCM docking onto the ORC-CDC6 ring (33, 35, 52, 58) (Fig. 5*C*). Threonine 466 in MCM5, which is mutated to isoleucine, resides within the AAA+ domain, near the MCM3•MCM5 ATPase site at the base of the pre-sensor 1 (PS1) pore loop that faces DNA (Fig. 5, *A*, *B*, and *D*). The other two mutations occur in MCM7 (Fig. 5, *A* and *B*). One of them, MCM7^G259A^, is located in a loop of the OB/Zn region, the conformation of which appears stabilized by contacts with E135 and I230. The second variant, MCM7^Y539C^, lies in a helix that interacts with MCM3, with the tyrosine stacking with nearby H543 and P547 (Fig. 5, *A*, *B*, and *E*). Both MCM7 substitutions may alter the local protein fold and weaken contacts between MCM7 and MCM4 or between MCM7 and MCM3.

To determine whether MGS mutations in MCM subunits interfere with replication initiation through the impediment of origin licensing, we purified mutant MCM complexes (each with a C-terminal GFP tag on MCM2) and examined their activities in the *in vitro* reconstituted origin licensing system. All MCM mutants could be isolated as an intact hexameric complex except for MCM7^Y539C^ (Fig. S3*A*); this mutation led to severe degradation and precipitation of MCM, indicating that this substitution compromised protein folding. When testing MCM-MGS variants in origin licensing assays, we found that they had distinct effects on MCM recruitment and loading (Figs. 5, *F* and *G* and Fig. S3*B*). While MCM7^G259A^ was as efficient as wildtype MCM in recruiting and loading MCM, the MCM3^Q761L^ and MCM5^T466I^ led to a strong decrease in MCM double hexamer formation (Fig. 5, *F* and *G*). Both mutants still supported efficient MCM recruitment, although it was reduced to 80% for MCM3^Q761L^ compared to wildtype MCM3 (Fig. 5, *F* and *G*). Since MCM3^Q761^ resides at the surface of the WH domain that interacts with ORC2 and CDC6 in OCCM-like assemblies, its mutation to leucine may challenge the initial recruitment of MCM to ORC-CDC6 and interfere with proper docking of MCM onto the ORC-CDC6 ring for MCM single hexamer deposition (Fig. 5, *A* and *C*). Indeed, the MCM3-WH region plays a critical role in the initial recruitment of MCM to ORC in budding yeast, as well as in a quality control pathway where it regulates ATPase activity of CDC6 and OCCM disassembly (52, 58). We note that our recruitment assay does not discriminate between conformationally distinct OCCM complexes that have been reported, *i.e.*, the semi-attached OCCM with MCM partially attached to ORC-CDC6, the pre-insertion OCCM before DNA insertion into MCM, and the canonical OCCM after DNA insertion into MCM, so it is possible that the relative abundance of these states is altered by MCM3^Q761L^ (33, 35, 55, 58). Nonetheless, our findings suggest that MCM3^Q761L^ is likely to stall MCM loading at the OCCM stage.

**Figure 6:**
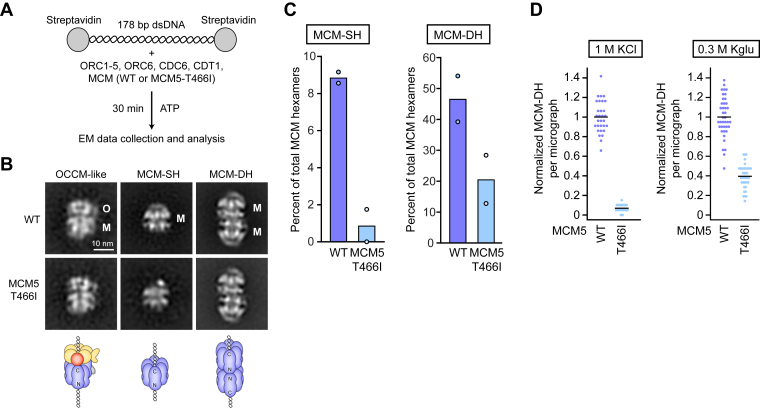
**The MGS mutation in MCM5 destabilizes MCM rings on DNA.***A*, experimental workflow for analyzing MCM loading intermediates. *B*, 2D EM class averages of negatively stained particles in MCM loading reactions done with WT MCM5 and MCM5^T466I^. *C*, quantification (by EM and 2D classification) of MCM-SH and MCM-DH particles in WT and MGS-mutant MCM loading reactions from two independent repeat experiments. *D*, quantification of MCM-DHs in the elutions of bead-based loading reactions after high-salt (1 M KCl) and low-salt (0.3 M potassium glutamate, Kglu) washes, normalized to reactions with WT MCM. Ten micrographs per reaction for each of four independent experiments were analyzed for 0.3 M Kglu-washed samples (total *n* = 40). The data for the 1 M KCl wash are replotted from Figure 5*G* for direct comparison. *Black* lines represent means.

As for MCM5^T466I^, the strong decrease in MCM double hexamer formation in standard MCM loading assays despite efficient MCM recruitment suggested that this mutation impedes a step during or after OCCM formation. Based on the location of MCM5^T466^ at the interface between MCM3 and MCM5 in proximity to the ATPase site, we hypothesized that this MGS variant might interfere with OCCM maturation into loaded MCM-SH and/or destabilize loaded MCM rings (Fig. 5*D*). To test this possibility, we visualized MCM loading reactions with wildtype MCM5 and MCM5^T466I^ by negative-stain electron microscopy (Fig. 6, *A* and *B*). As we observed previously (32), almost 50% of wildtype MCM hexamers were found in double hexamers after 30-min loading reactions with ATP, while ∼9% persisted as loaded MCM single hexamers (Fig. 6*C*). By contrast, the number of loaded MCM-SH was reduced nearly 10-fold when MCM5 was mutated, while that of MCM-DHs was less than half compared to wildtype MCM (Fig. 6*C*). OCCM-like particles were seen at low frequency in both conditions (Fig. 6*B*). Although these results are consistent with impaired MCM loading with MCM5^T466I^, we were surprised that the efficiency of MCM-DH formation in solution reactions was substantially higher than in our standard loading assays on beads (∼40% *versus* 10% of wildtype, Figs. 5*G* and 6*C*). We asked whether this difference was due to a lower stability of MCM on DNA that is exacerbated by the high-salt wash (1 M KCl) used in bead assays. Notably, reducing the salt concentration during the wash step of bead assays to 300 mM potassium glutamate increased the number of MCM-DH formed with MCM5^T466I^ compared to MCM^WT^, resulting in an MCM-DH formation efficiency similar to that observed in solution assays (Fig. 6*D*). These findings reinforce the notion that the MGS mutation in MCM5 weakens the loaded MCM ring, promoting dissociation of both MCM-SHs and MCM-DHs from DNA.

In summary, among the MGS mutations tested, only MCM7^G259A^ was able to support efficient origin licensing, suggesting that the MGS phenotype may arise from the impediment of origin firing or replisome activities in patients with this variant. The other MGS mutations compromise MCM recruitment, prevent proper MCM loading, or destabilize loaded MCM complexes.

## Discussion

Our systematic biochemical analyses of MGS mutations lends further credence to defects in DNA replication initiation – particularly during the MCM loading step – representing a major hallmark and driver of MGS disease pathogenesis. Although MGS mutations occur in multiple replication initiation factors, the extent to which their pathogenic effects reflect perturbations of replicative or non-replicative protein functions remains debated due to variable clinical manifestation of MGS and relatively mild replication defects observed in patient-derived cells (13, 14, 16, 19, 25, 26, 40, 59). Our findings help clarify this issue by establishing that all but one MGS-linked amino acid substitutions in ORC, CDC6, CDT1, and MCM directly reduce origin licensing efficiency. One notable exception is an MCM7 variant (G259A) that supports MCM loading to wildtype levels, suggesting the MGS phenotype associated with this mutation may arise from defects in MCM activation rather than MCM loading. Indeed, there is precedent linking MGS to impaired origin firing, as exemplified by mutations in the firing factors CDC45, GINS subunits, and DONSON (13, 60, 61, 62, 63, 64, 65, 66). Collectively, the mounting evidence underscores that Meier-Gorlin syndrome is fundamentally a disease of DNA replication initiation.

**Figure 7:**
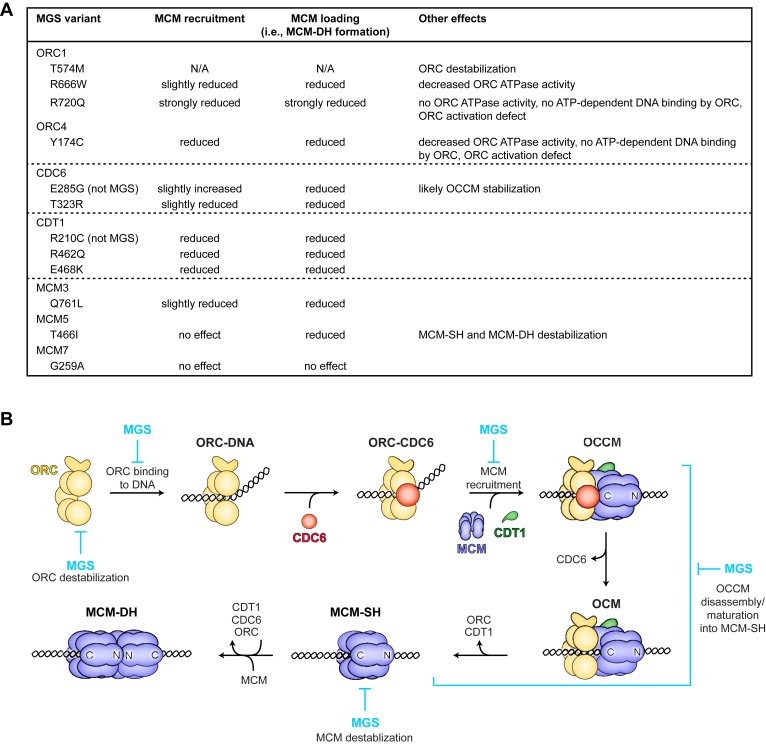
**Summary of origin licensing steps impeded by MGS mutations.***A*, list of origin licensing defects observed with MGS variant MCM loading factors used in this study. *B*, model of human origin licensing and steps affected in MGS.

MGS patients exhibit complex genotypes characterized by homozygous or compound heterozygous missense mutations, or combinations of loss-of-function and missense alleles. While MCM loading defects caused by loss-of-function variants can be easily rationalized, the molecular consequences of MGS missense mutations have been less clear. Our study reveals that the mechanisms underlying decreased origin licensing in the context of MGS missense variants are heterogeneous; in fact, even distinct missense mutations in the same licensing factor can impair different stages of MCM loading (Fig. 7). While some MGS mutations compromise protein folding and stability, potentially contributing to reduced protein levels in patient cells (13, 18), other variants accumulate as stable proteins but directly inhibit specific activities of MCM loading factors, thereby stalling MCM loading at discrete but distinct steps. For example, MGS substitutions in the ORC1•ORC4 ATPase site (ORC1^R720Q^ and ORC4^Y174C^) disrupt ORC activation and ATP-dependent DNA binding, thereby impeding ORC’s ability to recruit MCM to DNA. These results are consistent with the prediction that only ORC in the active conformation can stably engage DNA in its central channel (38, 48). By contrast, the ORC1^R666W^ variant in the CDC6•ORC1 ATPase site did not affect initiator DNA binding or MCM recruitment, but still significantly impaired MCM loading. Conspicuously, this mutant increased retention of ORC, CDC6, and possibly CDT1 during MCM recruitment, an outcome reminiscent of CDC6 ATPase mutants (35, 45, 46), suggesting a defect in ATP hydrolysis by CDC6. In yeast, Cdc6’s ATPase activity contributes to origin licensing quality control by disassembling non-productive MCM loading intermediates (52). Our findings are consistent with a similar function of CDC6 during human origin licensing and emphasize the non-redundant roles of ORC’s ATPase sites. The biochemical defects of ORC1 and ORC4 MGS variants also differ from those of ORC6 MGS mutants, which specifically inhibit directional loading of the second MCM hexamer during later stages of the licensing reaction (32). Thus, MGS mutations in ORC converge on a common outcome – impaired origin licensing – but through distinct mechanistic routes depending on the gene affected and the type of mutation.

MGS mutations in MCM subunits likewise displayed heterogeneous effects on origin licensing. MCM5^T466I^ was particularly intriguing as it reduced the stability of loaded MCM on DNA. Although purified MCM hexamers with MCM5^T466I^ appeared stable during purification and were efficiently recruited to DNA, both single and double MCM hexamer loading were strongly reduced. We propose that this variant, located at the MCM3/5 subunit interface, weakens inter-subunit contacts, thereby reducing the ability of MCM rings to withstand conformational strain associated with MCM ring closure during MCM-SH deposition, as well as to resist high-salt conditions. Additionally, substitution of MCM5^T466^ may perturb ATP binding or hydrolysis, given its proximity to the MCM5/3 ATPase center. MCM3^Q761L^ in the winged helix domain, on the other hand, impedes both MCM recruitment, albeit mildly, and loading. In yeast, this Mcm3 region establishes initial contacts with ORC and Cdc6, facilitating the docking of MCM onto the ORC-Cdc6 ring in the OCCM complex, and also stimulates ATP hydrolysis at the Cdc6•Orc1 site. In the human OCCM intermediate, this domain occupies a similar position as in the yeast (33, 35), indicating that these activities are likely conserved and could explain the licensing defects observed with MCM3^Q761L^. However, the MCM3 winged helix domain also interacts with the MCM2 ATPase domain in the free human MCM hexamer and appears to do so more efficiently with the MGS mutation, sterically hindering MCM ring closure (67). Consequently, the MCM loading defect associated with MCM3^Q761L^ likely arises from a combination of mechanisms. Future work will be needed to determine if MGS mutations in MCM also compromise specific origin firing steps in addition to origin licensing.

A surprising result from our work is the variability in the degree to which different MGS mutations impair MCM loading. While certain MGS variants of ORC and MCM supported only very low levels of MCM double hexamer loading (∼10% or less compared to wildtype), those of CDT1 had more moderate effects. It is difficult to directly correlate the extent of reduced origin licensing observed biochemically *in vitro* with clinical phenotype. However, it is noteworthy that the MGS variants of ORC1 and ORC4 are associated with more pronounced growth retardation than those of CDC6 or CDT1 (19), a trend that mirrors the number of MCM double hexamers loaded *in vitro*. A careful comparison of the phenotypes caused by different MGS variants in animal models of MGS, such as fly or zebrafish, could clarify whether both are indeed mechanistically linked (15, 68, 69, 70, 71, 72). Zebrafish models may be particularly useful in this context since they recapitulate the reduced organismal growth seen in MGS (15, 70, 71, 72).

Although our biochemical reconstitution system is powerful in gaining direct mechanistic insights into molecular events, it examines MCM loading in isolation and thus cannot fully replicate the complexities of the nuclear environment that inevitably modulate origin licensing. For one, the DNA substrate used is not chromatinized, which prevents analysis of MGS mutants mapping to protein regions that are involved in recruiting licensing factors to chromatin but not directly in loading events *per se*, including intrinsically disordered regions and the ORC1-BAH domain (70, 73, 74, 75). Future studies using chromatin substrates will be necessary to understand the molecular effects of these variants on origin licensing. Furthermore, our biochemical system does not account for non-replicative functions of MCM loading factors that may contribute to clinical phenotypes. For example, ORC1 is involved in centrosome homeostasis, and it has been proposed that deregulation of centrosome copy numbers by MGS variants in the IDR of ORC1 contributes to microcephaly and the more severe growth defects in these patients (25, 76). Finally, MCM plays a central role in both origin licensing and origin firing, and it is possible that inhibition of both events contributes to the MGS pathology. Testing this possibility directly will have to await the reconstitution of this replication initiation step with human proteins.

Collectively, our findings support models that MGS is fundamentally a disease of DNA replication initiation, but the precise defects are mutation- and gene-specific and arise from alterations of distinct biochemical activities. Appreciating these mechanistic differences not only deepens our understanding of the molecular pathology of MGS but also provides broader insights into fundamental principles of origin licensing and the link between dysregulated DNA replication and developmental syndromes.

## Experimental procedures

### Expression and purification of recombinant ORC1-5

Human ORC1-5 with an N-terminal truncation of ORC1 (ORC1^ΔN399^) and a natural variant in ORC4 (N78S) was expressed and purified using a multibaculovirus expression system as described in Yang *et al.* (32). Meier-Gorlin syndrome (MGS) mutations in ORC1ΔN (T574M, R666W, R720Q) and ORC4 (Y174C) were introduced by site-directed mutagenesis. Wildtype ORC1-5 was cloned into a single multibac construct, ORC1-MGS mutants were co-expressed with a multibac construct encoding ORC2-5, and the ORC4-MGS mutant was co-expressed individually with ORC1ΔN, ORC5, and a multibac construct encoding ORC2 and ORC3. An N-terminal 6xHis-TEV purification tag was included in all ORC1 constructs, while MBP-TEV was added to the N-terminus of ORC4. For expression and purification of ORC2-5, the 6xHis tag was moved to the N-terminus of ORC2.

ORC was expressed in High Five cells by 48-h infection with P2-amplified baculovirus as done previously (32). Cell pellets were resuspended in 35 ml lysis buffer (50 mM Tris-HCl pH 7.8, 300 mM KCl, 50 mM imidazole, 10% glycerol, 200 μM PMSF, 1 μg/ml leupeptin and 1 mM β-ME) per liter culture and lysed by sonication, after which the lysate was clarified by ammonium sulfate precipitation and two rounds of ultracentrifugation. ORC was purified by nickel-affinity chromatography on a 5 ml HisTrap HP column (Cytiva) and eluted with 250 mM imidazole in 50 mM Tris-HCl pH 7.8, 300 mM KCl, 10% glycerol, and 1 mM β-ME. A second affinity purification step was done on a 7 to 10 ml amylose column (New England Biolabs) and ORC was eluted with 20 mM maltose in 50 mM Tris-HCl pH 7.8, 300 mM KCl, 10% glycerol, and 1 mM β-ME. Affinity tags were cleaved by 6xHis-TEV protease digestion overnight at 4 °C, followed by another nickel-affinity step to remove uncleaved protein and TEV using a 5 ml HisTrap HP column (Cytiva). ORC was further purified by size exclusion chromatography on HiPrep 16/60 Sephacryl S-400 HR or Superose 6 Increase 10/300 Gl columns (Cytiva) equilibrated in 25 mM HEPES pH 7.6, 500 mM potassium glutamate, 10% glycerol, and 1 mM DTT. ORC was then concentrated in 30 K Amicon Ultra-15 concentrators (Millipore), aliquoted, and flash frozen in liquid nitrogen for storage at −80 °C.

### Expression and purification of recombinant ORC6

Human ORC6 was expressed in BL21 RIL *E. coli* cells as a 6xHis-TEV fusion and purified as described in detail in reference (32). Briefly, ORC6 expression was induced in 2 L of culture by addition of 0.5 mM IPTG at an OD_600 nm_ of 0.4 to 0.6 for ∼18 h at 16 °C. Cell pellets were resuspended in ∼60 ml lysis buffer (50 mM Tris-HCl pH 8, 800 mM KCl, 30 mM imidazole, 10% glycerol, 200 μM PMSF, 1 μg/ml leupeptin, 1 mM β-ME). After sonication and lysate clarification by centrifugation, ORC6 was purified by nickel-affinity chromatography on a 5 ml HisTrap HP column (Cytiva) and eluted with 250 mM imidazole in 50 mM Tris-HCl pH 8, 150 mM KCl, 10% glycerol, and 1 mM β-ME after a 200 ml wash with lysis buffer. The 6xHis tag was removed by overnight cleavage with 6xHis-TEV protease during dialysis against 50 mM Tris-HCl pH 8, 150 mM KCl, 30 mM imidazole, 10% glycerol, and 1 mM β-ME and passage of the protein solution over a 5 ml HisTrap HP column (Cytiva). ORC6 was then loaded onto a HiLoad 16/60 Superdex 75 pg column (Cytiva) equilibrated in 50 mM Tris-HCl pH 8, 150 mM KCl, 10% glycerol, and 1 mM DTT. Peak fractions were pooled, concentrated, aliquoted, flash-frozen in liquid nitrogen, and stored at −80 °C.

### Expression and purification of recombinant CDC6

CDC6 was expressed as full-length protein in High Five insect cells as an N-terminal 6xHis-MBP fusion as previously described (32). Point mutations T323R (MGS mutant) and E285G (ATPase-defective mutant) were generated by site-directed mutagenesis and verified by DNA sequencing. All CDC6 constructs were purified after 48-h baculovirus infection. High Five cells were resuspended and sonicated in 35 ml lysis buffer (50 mM Tris-HCl pH 7.8, 300 mM KCl, 50 mM imidazole, 10% glycerol, 200 μM PMSF, 1 μg/ml leupeptin, 1 mM β-ME) per liter of culture. After two rounds of ultracentrifugation and ammonium sulfate precipitation, the supernatant was loaded onto a 5 ml HisTrap HP Nickel-affinity chromatography column (Cytiva), which was washed with 60 ml lysis buffer. CDC6 was eluted with 250 mM imidazole in lysis buffer without protease inhibitors. A second affinity purification step was done on a 5 to 10 ml amylose column (New England Biolabs) and the protein eluted in wash buffer (50 mM Tris-HCl pH 7.8, 300 mM KCl, 10% glycerol, 1 mM β-ME) supplemented with 20 mM maltose. After size exclusion chromatography on a HiLoad 16/600 Superdex 200 pg column (Cytiva), peak CDC6 fractions were pooled and digested overnight with 6xHis-tagged TEV protease, followed by another nickel affinity step using a 5 ml HisTrap HP column (Cytiva). Cleaved CDC6 was finally purified by gel filtration chromatography on a HiLoad Superdex 200 pg column (Cytiva) or a 10/300 Gl Superdex 200 column (Cytiva) in 50 mM Tris-HCl pH 7.8, 300 mM KCl, 10% glycerol, 1 mM DTT and then concentrated, aliquoted, and flash-frozen in liquid nitrogen.

### Expression and purification of recombinant CDT1

Wildtype and mutant, full-length human CDT1 with an N-terminal 6xHis-MBP-TEV tag were expressed in baculovirus-infected High Five cells for 48 h. MGS mutants (R462Q, R453W, and E468K) and R210C were introduced by site-directed mutagenesis into CDT1 that had been cloned into a LIC-compatible pFastBac vector (77). For purification, insect cells were harvested and resuspended in 60 ml lysis buffer (50 mM Tris-HCl pH 7.8, 1 M NaCl, 30 mM imidazole, 10% glycerol, 200 μM PMSF, 5 mM β-ME, 1 μg/ml leupeptin). After sonication, ultracentrifugation and ammonium sulfate precipitation as described previously (32), CDT1 was purified using nickel affinity chromatography on a 5 ml HisTrap HP column (Cytiva) with a 200 ml wash with lysis buffer and a 50 ml wash with low-salt buffer (50 mM Tris-HCl pH 7.8, 300 mM KCl, 30 mM imidazole, 10% glycerol, 5 mM β-ME). CDT1 was eluted with 250 mM imidazole in low-salt buffer directly onto a 5 ml HiTrap Q HP ion exchange column (Cytiva). The flow-through was further purified on a 5 ml amylose column (New England Biolabs) that was washed with 50 ml wash buffer (50 ml Tris-HCl pH 7.8, 300 mM KCl, 10% glycerol, 5 mM β-ME) and eluted with 20 mM maltose in wash buffer. 6xHis-MBP-*Hs*CDT1 was loaded onto a HiLoad 16/600 Superdex 200 pg column (Cytiva) equilibrated in 50 mM Tris-HCl pH 7.8, 150 mM KCl, 10% glycerol, and 1 mM DTT. The protein was concentrated in a 30 K Amicon Ultra-15 concentrator (Millipore), aliquoted, flash frozen in liquid nitrogen, and stored at −80 °C. Purifications of MGS mutant R453W repeatedly resulted in poor yields and largely degraded protein, suggesting the mutation destabilized the protein fold; thus, this CDT1 mutant could not be analyzed biochemically.

Wildtype truncated human CDT1 (CDT1ΔN, amino acids 167–546) was expressed with an N-terminal 6xHis tag in BL21 RIL *E. coli* cells and purified as described previously (32). The cell lysate was clarified by centrifugation at 23,426*g* and purified by nickel-affinity and ion exchange columns as described above. The N-terminal 6xHis tag was removed by digestion with 6xHis-tagged TEV protease during overnight dialysis into 50 mM Tris-HCl pH 7.8, 150 mM KCl, 10% glycerol, 5 mM β-ME and subsequent nickel affinity chromatography. The flow-through was concentrated and loaded onto a HiLoad 16/600 Superdex 200 pg column (Cytiva). Protein peak fractions were concentrated, aliquoted, and flash frozen for storage at −80 °C. Buffers were the same as described for the purification of CDT1 mutants.

### Expression and purification of recombinant MCM2-7

MCM2-7 was reconstituted in insect cells by co-expressing the six subunits using the BioBricks MultiBac expression system. One of the baculoviruses encoded 6xHis-TEV-MCM4 (natural variant L650M), MCM6, and MCM2 (with or without a C-terminal msfGFP tag), and the other encoded MCM5, MBP-TEV-MCM3, and MCM7. MGS mutations MCM5^T466I^, MCM3^Q761L^, MCM7^G259A^, and MCM7^Y539C^ were introduced by site-directed mutagenesis. For MCM purification (as described in (32)), High Five cells were harvested 48 h after infection with P2-amplified multi-baculoviruses and resuspended and sonicated in 35 ml lysis buffer (50 mM HEPES-KOH pH 7.5, 300 mM potassium acetate, 10% glycerol, 30 mM imidazole, 1 mM β-ME, 1 μg/ml leupeptin, 200 μM PMSF) per liter culture. Cell lysates were clarified by ultracentrifugation and ammonium sulfate precipitation, and MCM purified by a nickel affinity (5 ml HisTrap HP column, Cytiva), amylose affinity (6 ml amylose column, New England Biolabs), and size exclusion (Superose 6 Increase 10/300 Gl column, Cytiva) chromatography. Prior to gel filtration, the purification tags were removed by TEV cleavage as done previously (32). Purified MCM (in 25 mM HEPES-KOH pH 7.5, 300 mM potassium acetate, 10% glycerol, 1 mM DTT) was concentrated and flash-frozen in liquid nitrogen for long-term storage. The MCM7^Y539C^ exhibited degradation during purification and precipitated during overnight TEV protease digestion, suggesting that the mutation compromised the stability and integrity of MCM2-7 complex. Consequently, MCM7^Y539C^ was not further studied.

### MCM2-7 recruitment and loading assay

MCM recruitment and loading were analyzed using a 178 bp biotinylated DNA fragment (for sequence, see (32)) that was coupled to Dynabeads MyOne Streptavidin T1 beads (Thermo Fisher Scientific) (32). 3 pmol of biotinylated DNA per reaction were bound to 10 μl magnetic beads and any free DNA ends blocked by adding streptavidin (3 pmol per reaction, Sigma-Aldrich). Each 40 μl-reaction contained 60 nM ORC1-5 (with ORC1ΔN), 60 nM ORC6, 60 nM CDC6, 120 nM CDT1 (CDT1ΔN except for reactions analyzing CDT1 mutants, where full-length, 6xHis-MBP-tagged protein was used), and 120 nM MCM2-7-GFP (GFP on C-terminus of MCM2) in low-salt buffer (25 mM HEPES-KOH pH 7.6, 300 mM potassium glutamate, 10 mM magnesium acetate, 10% glycerol, 0.01% NP-40, 1 mM DTT) with 1 mM ATP (for loading reactions) or 1 mM ATPγS (for recruitment reactions). Note that N-terminally truncated ORC1 and CDT1 (ORC1ΔN and CDT1ΔN) support MCM recruitment and loading with the same efficiency as full-length proteins (32). Reactions were incubated at 37°C for 30 min prior to washing steps. For MCM loading, beads were washed once with 1 ml high-salt buffer (25 mM HEPES-KOH pH 7.6, 1 M KCl, 10 mM magnesium acetate, 10% glycerol, 0.01% NP-40, 1 mM DTT, 1 mM ATP), and once with 1 ml low-salt buffer with 1 mM ATP. For MCM recruitment, beads were washed twice with low-salt wash (without ATPγS). DNA-bound proteins were eluted by digestion with 500 units MNase (New England Biolabs) per reaction for 10 min at 37 °C in 25 mM HEPES pH 7.6, 300 mM KCl, 5 mM CaCl_2_, 10% glycerol, 1 mM DTT with 1 mM ATP (for loading reactions) or 1 mM ATPγS (for recruitment reactions), followed by GFP fluorescence measurements in eluates using a PHERAstar FSX plate reader (BMG Labtech) with excitation and emission at 485 nm and 520 nm, respectively. A minimum of three independent experiments were performed and the fluorescence values normalized to the average reading from reactions with wildtype proteins. MCM loading efficiency was further assessed by counting MCM-DH numbers by negative-stain EM (see below).

### ATPase assay

Steady-state ATP hydrolysis by human ORC1-5 and ORC2-5 was measured as done previously for *Drosophila* ORC using the ATP/NADH-coupled ATPase assay (28, 78). Reactions (in 50 μl) contained 1 μM wildtype or MGS-mutant *Hs*ORC1-5, 4 mM phosphoenolpyruvate, 0.3 mM NADH, 1.2 to 2 units/ml pyruvate kinase, 1.8 to 2.8 units/ml lactic dehydrogenase (from rabbit muscle, Sigma Aldrich), 0.1 mg/ml BSA, 25 mM HEPES-KOH (pH 7.6), 300 mM potassium glutamate, 10% glycerol, 1 mM DTT, 10 mM magnesium acetate, 0.01% NP-40. ATP was titrated as 2.5-fold serial dilutions from 0.13 μM to 0.5 mM. ATP hydrolysis was measured as decrease in NADH absorbance at 340 nm in a PHERAstar FSX plate reader (BMG Labtech) at 37 °C for 2 h every 60 s. Hydrolysis rates were determined from the linear portions of the NADH consumption curves and plotted as a function of ATP concentration. Data points from three independent experiments were fit to the Michaelis-Menten equation using GraphPad Prism to determine k_cat_ values and standard errors of fits.

## Fluorescence anisotropy DNA binding assay

DNA binding assays for wildtype and MGS-mutant *Hs*ORC1-5 were performed as described previously for the *Drosophila* complex (28, 48). 40 bp fluorescein labeled dsDNA (annealed using 5′-FluorT/TTTTGAAAAGCAAGCATAAAAGATCTAAACATAAAA TCTG-3′ and 5′-CAGATTTTATGTTTAGATCTTTT ATGCTTGCTT TTCAAAA-3′) at 1 nM was incubated with increasing concentrations of wildtype or MGS-mutant ORC1-5 (from 38 nM to 2.5 μM) in 25 mM HEPES-KOH (pH 7.6), 300 mM potassium glutamate, 10% glycerol, 1 mM DTT, 10 mM magnesium acetate, 0.01% NP-40, and 0 or 1 mM ATP for 30 min at 22 °C. 20 μl of each binding reaction were transferred into a 384-well plate and anisotropy measured in a PHERAstar FSX plate reader (BMG Labtech). Data points from three independent experiments for each ORC1-5 complex were fit to the Hill binding model to calculate apparent dissociation constants (K_d,app_) in GraphPad Prism.

### Negative-stain EM

#### MCM2-7 loading reaction eluates

4 μl of reaction eluates from canonical loading reactions were applied to glow-discharged continuous carbon grids (Ted Pella) and incubated for 1 min. Grids were stained with three drops of 40 μl 2% uranyl acetate for 10 s each. Excess uranyl acetate was blotted away after a 30-s incubation with stain. EM grids were imaged in a Talos L120C transmission electron microscope at 120 kV and a magnification of 45 kx. Ten micrographs from two to three different grid squares were manually recorded per reaction, and the number of MCM2-7 double hexamer particles counted manually in each micrograph. The number of MCM2-7 double hexamers per micrograph was normalized to the average in reactions with wildtype proteins and ATP. Statistical significance was calculated using the two-way ANOVA test with Tukey *post hoc* analysis in GraphPad Prism as done previously (32).

#### MCM2-7 loading intermediates

120 nM ORC1-5 (with ORC1ΔN), ORC6, CDC6, CDT1ΔN, and GFP-MCM2-7 (WT or MCM5^T466I^) were mixed with 180 nM biotinylated 178 bp DNA and 360 nM streptavidin in low-salt buffer with 1 mM ATP. Before mixing, MCM2-7 alone was incubated at 37 °C for 20 min to dissociate MCM2-7 dimers (32). Loading reactions were incubated at 37 °C for 30 min, placed on ice for 10 s, and 4 μl were immediately applied to EM grids. After a 10-s incubation, grids were stained sequentially on three 40 μl drops of 2% uranyl acetate for 10 s each, followed by a final 30-s incubation. Datasets from independently prepared reactions were collected at 45 kx magnification with around 120 micrographs per dataset. Particles were automatically picked using GAUTOMATCH (K. Zhang, MRC-LMB, Cambridge), extracted from micrographs after phase-flipping and CTF determination with GCTF (79), and analyzed by 2D classification in RELION 4.0.1 (80).

#### ORC1 conformations of wildtype and MGS-mutant ORC

Wildtype and MGS-mutant ORC1-5 were diluted to 80 nM in 25 mM HEPES-KOH (pH 7.6), 10 mM magnesium acetate, 0.3 M potassium glutamate, 10% glycerol, 1 mM DTT, and 1 mM ATP. 4 μl of the dilution were adsorbed to a glow-discharged negative-stain EM grid for 30 s and then stained with 2% uranyl acetate. Electron micrographs were collected at 73 kx magnification in a Talos L120C transmission electron microscope using SerialEM (81) and then imported into CryoSPARC v4.2.1 (82) for particle picking and 2D classification. For each dataset, 60,000 or more ORC particles were obtained and sorted into 150 classes. Class averages were compared to 2D projections of active and autoinhibited/inactive ORC structures (PDB 4XGC (38) and PDB 7JK6 (28)). Since only a subset of class averages and projections differ between both ORC conformations, the number of active particles is likely higher than the one reported here. Two datasets from grids prepared independently were collected for each ORC construct and processed separately.

### Mass photometry

Mass photometry measurements were performed at room temperature using a TwoMP mass photometer (Refeyn). Wildtype and MGS variants of ORC were diluted to 200 nM in a low-salt buffer (25 mM HEPES-KOH pH 7.6, 300 mM potassium glutamate, 10 mM magnesium acetate, 10% glycerol, 1 mM DTT) containing 1 mM ATP. 18 μl of low-salt buffer was added to a clean glass coverslip with a six-well silicone gasket and used to set the focus. Subsequently, 2 μl of the protein sample was added and mixed with buffer to a final concentration of 20 nM ORC. One-minute movies were recorded with the AcquireMP (AMP) software and analyzed using DiscoverMP (DMP) software. Mass photometry histograms were fitted to Gaussian distributions to calculate the counts corresponding to ORC monomer and dimer peaks, enabling quantification of monomer-dimer ratios. Molecular masses were determined from contrast values using the Contrast-to-Mass (CTM) calibration. For mass calibration, bovine serum albumin (BSA, monomer 66 kDa, dimer 132 kDa), apoferritin (480 kDa), and thyroglobulin (660 kDa) were used.

### Structure analysis

PyMOL (The PyMOL Molecular Graphics System, Schrödinger) was used for structure visualization and figure generation.

### Statistics and reproducibility

All MCM loading assay experiments, including SDS-PAGE gels and fluorescence measurements, as well as ORC ATPase and DNA binding assays were performed in at least three independent replicates with consistent results. The means and standard deviations from at least three independent experiments are plotted. To quantify MCM2-7 double hexamers in loading assay eluates by negative-stain EM, at least three independent replicates were analyzed, with a minimum of ten micrographs per replicate (n ≥ 30). Statistical significance of mean values was assessed using two-way ANOVA followed by Tukey’s multiple comparison test. For negative-stain EM analyses of ORC conformations and MCM2-7 loading intermediates, two independent experiments were conducted, with 100 to 150 micrographs analyzed per experiment and consistent results obtained. The ORC1-5 stability assay was carried out using two independently purified protein samples. Mass photometry experiments were conducted at least twice independently.

## Data availability

Data related to this manuscript are included in the main text and supporting information. Materials are available upon reasonable request from the corresponding author.

## Supporting information

This article contains supporting information.

## Declaration of Generative AI and AI-Assisted Technologies in the Writing Process

During the preparation of this work, the author(s) used “ChatGPT” in order to edit sections of the manuscript to improve clarity. After using this tool/service, the author(s) reviewed and edited the content as needed and take(s) full responsibility for the content of the publication.

## Conflict of interest

F. B. is a member of the Yale Cancer Center. The authors declare that they have no conflicts of interest with the contents of this article.

## Supporting information

Supporting informtion

## References

[1] Remus D., Beuron F., Tolun G., Griffith J.D., Morris E.P., Diffley J.F. (2009). Concerted loading of Mcm2-7 double hexamers around DNA during DNA replication origin licensing. Cell.

[2] Evrin C., Clarke P., Zech J., Lurz R., Sun J., Uhle S. (2009). A double-hexameric MCM2-7 complex is loaded onto origin DNA during licensing of eukaryotic DNA replication. Proc. Natl. Acad. Sci. U. S. A..

[3] Gambus A., Khoudoli G.A., Jones R.C., Blow J.J. (2011). MCM2-7 form double hexamers at licensed origins in Xenopus egg extract. J. Biol. Chem..

[4] Bleichert F. (2019). Mechanisms of replication origin licensing: a structural perspective. Curr. Opin. Struct. Biol..

[5] Costa A., Diffley J.F.X. (2022). The initiation of eukaryotic DNA replication. Annu. Rev. Biochem..

[6] Attali I., Botchan M.R., Berger J.M. (2021). Structural mechanisms for replicating DNA in eukaryotes. Annu. Rev. Biochem..

[7] Nordman J., Orr-Weaver T.L. (2012). Regulation of DNA replication during development. Development.

[8] Siddiqui K., On K.F., Diffley J.F. (2013). Regulating DNA replication in eukarya. Cold Spring Harb. Perspect. Biol..

[9] Moiseeva T.N., Bakkenist C.J. (2018). Regulation of the initiation of DNA replication in human cells. DNA Repair (Amst).

[10] Klingseisen A., Jackson A.P. (2011). Mechanisms and pathways of growth failure in primordial dwarfism. Genes Dev..

[11] Schmit M., Bielinsky A.K. (2021). Congenital diseases of DNA replication: clinical phenotypes and molecular mechanisms. Int. J. Mol. Sci..

[12] Bellelli R., Boulton S.J. (2021). Spotlight on the replisome: aetiology of DNA replication-associated genetic diseases. Trends Genet..

[13] Nielsen-Dandoroff E., Ruegg M.S.G., Bicknell L.S. (2023). The expanding genetic and clinical landscape associated with Meier-Gorlin syndrome. Eur. J. Hum. Genet..

[14] Bicknell L.S., Bongers E.M., Leitch A., Brown S., Schoots J., Harley M.E. (2011). Mutations in the pre-replication complex cause Meier-Gorlin syndrome. Nat. Genet..

[15] Bicknell L.S., Walker S., Klingseisen A., Stiff T., Leitch A., Kerzendorfer C. (2011). Mutations in ORC1, encoding the largest subunit of the origin recognition complex, cause microcephalic primordial dwarfism resembling Meier-Gorlin syndrome. Nat. Genet..

[16] Guernsey D.L., Matsuoka M., Jiang H., Evans S., Macgillivray C., Nightingale M. (2011). Mutations in origin recognition complex gene ORC4 cause Meier-Gorlin syndrome. Nat. Genet..

[17] Vetro A., Savasta S., Russo Raucci A., Cerqua C., Sartori G., Limongelli I. (2017). MCM5: a new actor in the link between DNA replication and Meier-Gorlin syndrome. Eur. J. Hum. Genet..

[18] Knapp K.M., Jenkins D.E., Sullivan R., Harms F.L., von Elsner L., Ockeloen C.W. (2021). MCM complex members MCM3 and MCM7 are associated with a phenotypic spectrum from Meier-Gorlin syndrome to lipodystrophy and adrenal insufficiency. Eur. J. Hum. Genet..

[19] de Munnik S.A., Bicknell L.S., Aftimos S., Al-Aama J.Y., van Bever Y., Bober M.B. (2012). Meier-Gorlin syndrome genotype-phenotype studies: 35 individuals with pre-replication complex gene mutations and 10 without molecular diagnosis. Eur. J. Hum. Genet..

[20] Pak D.T., Pflumm M., Chesnokov I., Huang D.W., Kellum R., Marr J. (1997). Association of the origin recognition complex with heterochromatin and HP1 in higher eukaryotes. Cell.

[21] Prasanth S.G., Prasanth K.V., Stillman B. (2002). Orc6 involved in DNA replication, chromosome segregation, and cytokinesis. Science.

[22] Chesnokov I.N., Chesnokova O.N., Botchan M. (2003). A cytokinetic function of Drosophila ORC6 protein resides in-a domain distinct from its replication activity. Proc. Natl. Acad. Sci. U. S. A..

[23] Prasanth S.G., Prasanth K.V., Siddiqui K., Spector D.L., Stillman B. (2004). Human Orc2 localizes to centrosomes, centromeres and heterochromatin during chromosome inheritance. EMBO J..

[24] Popova V.V., Brechalov A.V., Georgieva S.G., Kopytova D.V. (2018). Nonreplicative functions of the origin recognition complex. Nucleus.

[25] Hossain M., Stillman B. (2012). Meier-Gorlin syndrome mutations disrupt an Orc1 CDK inhibitory domain and cause centrosome reduplication. Genes Dev..

[26] Stiff T., Alagoz M., Alcantara D., Outwin E., Brunner H.G., Bongers E.M. (2013). Deficiency in origin licensing proteins impairs cilia formation: implications for the aetiology of Meier-Gorlin syndrome. PLoS Genet..

[27] Bell S.P., Stillman B. (1992). ATP-dependent recognition of eukaryotic origins of DNA replication by a multiprotein complex. Nature.

[28] Schmidt J.M., Bleichert F. (2020). Structural mechanism for replication origin binding and remodeling by a metazoan origin recognition complex and its co-loader Cdc6. Nat. Commun..

[29] Sun J., Evrin C., Samel S.A., Fernandez-Cid A., Riera A., Kawakami H. (2013). Cryo-EM structure of a helicase loading intermediate containing ORC-Cdc6-Cdt1-MCM2-7 bound to DNA. Nat. Struct. Mol. Biol..

[30] Samel S.A., Fernandez-Cid A., Sun J., Riera A., Tognetti S., Herrera M.C. (2014). A unique DNA entry gate serves for regulated loading of the eukaryotic replicative helicase MCM2-7 onto DNA. Genes Dev..

[31] Ticau S., Friedman L.J., Champasa K., Correa I.R., Gelles J., Bell S.P. (2017). Mechanism and timing of Mcm2-7 ring closure during DNA replication origin licensing. Nat. Struct. Mol. Biol..

[32] Yang R., Hunker O., Wise M., Bleichert F. (2024). Multiple mechanisms for licensing human replication origins. Nature.

[33] Weissmann F., Greiwe J.F., Puhringer T., Eastwood E.L., Couves E.C., Miller T.C.R. (2024). MCM double hexamer loading visualized with human proteins. Nature.

[34] Li N., Zhai Y., Zhang Y., Li W., Yang M., Lei J. (2015). Structure of the eukaryotic MCM complex at 3.8 A. Nature.

[35] Wells J.N., Edwardes L.V., Leber V., Allyjaun S., Peach M., Tomkins J. (2025). Reconstitution of human DNA licensing and the structural and functional analysis of key intermediates. Nat. Commun..

[36] Neuwald A.F., Aravind L., Spouge J.L., Koonin E.V. (1999). AAA+: a class of chaperone-like ATPases associated with the assembly, operation, and disassembly of protein complexes. Genome Res..

[37] Iyer L.M., Leipe D.D., Koonin E.V., Aravind L. (2004). Evolutionary history and higher order classification of AAA+ ATPases. J. Struct. Biol..

[38] Bleichert F., Botchan M.R., Berger J.M. (2015). Crystal structure of the eukaryotic origin recognition complex. Nature.

[39] Vashee S., Simancek P., Challberg M.D., Kelly T.J. (2001). Assembly of the human origin recognition complex. J. Biol. Chem..

[40] Bleichert F., Balasov M., Chesnokov I., Nogales E., Botchan M.R., Berger J.M. (2013). A Meier-Gorlin syndrome mutation in a conserved C-terminal helix of Orc6 impedes origin recognition complex formation. eLife.

[41] Bowers J.L., Randell J.C., Chen S., Bell S.P. (2004). ATP hydrolysis by ORC catalyzes reiterative Mcm2-7 assembly at a defined origin of replication. Mol. Cell.

[42] Speck C., Stillman B. (2007). Cdc6 ATPase activity regulates ORC x Cdc6 stability and the selection of specific DNA sequences as origins of DNA replication. J. Biol. Chem..

[43] Randell J.C., Bowers J.L., Rodriguez H.K., Bell S.P. (2006). Sequential ATP hydrolysis by Cdc6 and ORC directs loading of the Mcm2-7 helicase. Mol. Cell.

[44] Tocilj A., On K.F., Yuan Z., Sun J., Elkayam E., Li H. (2017). Structure of the active form of human origin recognition complex and its ATPase motor module. eLife.

[45] Coster G., Frigola J., Beuron F., Morris E.P., Diffley J.F. (2014). Origin licensing requires ATP binding and hydrolysis by the MCM replicative helicase. Mol. Cell.

[46] Kang S., Warner M.D., Bell S.P. (2014). Multiple functions for Mcm2-7 ATPase motifs during replication initiation. Mol. Cell.

[47] Li N., Lam W.H., Zhai Y., Cheng J., Cheng E., Zhao Y. (2018). Structure of the origin recognition complex bound to DNA replication origin. Nature.

[48] Bleichert F., Leitner A., Aebersold R., Botchan M.R., Berger J.M. (2018). Conformational control and DNA-binding mechanism of the metazoan origin recognition complex. Proc. Natl. Acad. Sci. U. S. A..

[49] Jaremko M.J., On K.F., Thomas D.R., Stillman B., Joshua-Tor L. (2020). The dynamic nature of the human origin recognition complex revealed through five cryoEM structures. eLife.

[50] Perkins G., Diffley J.F. (1998). Nucleotide-dependent prereplicative complex assembly by Cdc6p, a homolog of eukaryotic and prokaryotic clamp-loaders. Mol. Cell.

[51] Speck C., Chen Z., Li H., Stillman B. (2005). ATPase-dependent cooperative binding of ORC and Cdc6 to origin DNA. Nat. Struct. Mol. Biol..

[52] Frigola J., Remus D., Mehanna A., Diffley J.F. (2013). ATPase-dependent quality control of DNA replication origin licensing. Nature.

[53] Chang F., Riera A., Evrin C., Sun J., Li H., Speck C. (2015). Cdc6 ATPase activity disengages Cdc6 from the pre-replicative complex to promote DNA replication. eLife.

[54] Pozo P.N., Matson J.P., Cole Y., Kedziora K.M., Grant G.D., Temple B. (2018). Cdt1 variants reveal unanticipated aspects of interactions with cyclin/CDK and MCM important for normal genome replication. Mol. Biol. Cell.

[55] Yuan Z., Riera A., Bai L., Sun J., Nandi S., Spanos C. (2017). Structural basis of Mcm2-7 replicative helicase loading by ORC-Cdc6 and Cdt1. Nat. Struct. Mol. Biol..

[56] Whittaker A.J., Royzman I., Orr-Weaver T.L. (2000). Drosophila double parked: a conserved, essential replication protein that colocalizes with the origin recognition complex and links DNA replication with mitosis and the down-regulation of S phase transcripts. Genes Dev..

[57] You Z., Ode K.L., Shindo M., Takisawa H., Masai H. (2016). Characterization of conserved arginine residues on Cdt1 that affect licensing activity and interaction with Geminin or Mcm complex. Cell Cycle.

[58] Yuan Z., Schneider S., Dodd T., Riera A., Bai L., Yan C. (2020). Structural mechanism of helicase loading onto replication origin DNA by ORC-Cdc6. Proc. Natl. Acad. Sci. U. S. A..

[59] Tingler M., Philipp M., Burkhalter M.D. (2022). DNA replication proteins in primary microcephaly syndromes. Biol. Cell.

[60] Fenwick A.L., Kliszczak M., Cooper F., Murray J., Sanchez-Pulido L., Twigg S.R. (2016). Mutations in CDC45, encoding an essential component of the pre-initiation complex, cause meier-gorlin syndrome and craniosynostosis. Am. J. Hum. Genet..

[61] Nabais Sa M.J., Miller K.A., McQuaid M., Koelling N., Wilkie A.O.M., Wurtele H. (2022). Biallelic GINS2 variant p.(Arg114Leu) causes Meier-Gorlin syndrome with craniosynostosis. J. Med. Genet..

[62] McQuaid M.E., Ahmed K., Tran S., Rousseau J., Shaheen R., Kernohan K.D. (2022). Hypomorphic GINS3 variants alter DNA replication and cause Meier-Gorlin syndrome. JCI Insight.

[63] Knapp K.M., Sullivan R., Murray J., Gimenez G., Arn P., D'Souza P. (2020). Linked-read genome sequencing identifies biallelic pathogenic variants in DONSON as a novel cause of Meier-Gorlin syndrome. J. Med. Genet..

[64] Karaca E., Posey J.E., Bostwick B., Liu P., Gezdirici A., Yesil G. (2019). Biallelic and De Novo Variants in DONSON Reveal a Clinical Spectrum of Cell Cycle-opathies with Microcephaly, Dwarfism and Skeletal Abnormalities. Am. J. Med. Genet. A..

[65] Nerakh G., Vineeth V.S., Tallapaka K., Nair L., Dalal A., Aggarwal S. (2022). Microcephalic primordial dwarfism with predominant Meier-Gorlin phenotype, ichthyosis, and multiple joint deformities-further expansion of DONSON cell cycle-opathy phenotypic spectrum. Am. J. Med. Genet. A..

[66] Reynolds J.J., Bicknell L.S., Carroll P., Higgs M.R., Shaheen R., Murray J.E. (2017). Mutations in DONSON disrupt replication fork stability and cause microcephalic dwarfism. Nat. Genet..

[67] Liu Y., Yang M., Lu P., Gao H., He M., Wang Y. (2025). Cryo-EM structure of DNA-unbound human MCM2–7 complex reveals new disease-relevant regulation. bioRxiv.

[68] Balasov M., Akhmetova K., Chesnokov I. (2015). Drosophila model of Meier-Gorlin syndrome based on the mutation in a conserved C-Terminal domain of Orc6. Am. J. Med. Genet. A..

[69] McDaniel S.L., Hollatz A.J., Branstad A.M., Gaskill M.M., Fox C.A., Harrison M.M. (2020). Tissue-specific DNA replication defects in Drosophila melanogaster caused by a Meier-Gorlin Syndrome Mutation in Orc4. Genetics.

[70] Kuo A.J., Song J., Cheung P., Ishibe-Murakami S., Yamazoe S., Chen J.K. (2012). The BAH domain of ORC1 links H4K20me2 to DNA replication licensing and Meier-Gorlin syndrome. Nature.

[71] Yao L.K., Chen J., Wu X.T., Jia S.J., Meng A.M. (2017). Zebrafish hypomorphic mutation causes Meier-Gorlin syndrome-like phenotype. Hum. Mol. Genet..

[72] Maerz L.D., Tena T.C., Gerhards J., Donow C., Jeggo P.A., Philipp M. (2019). Analysis of cilia dysfunction phenotypes in zebrafish embryos depleted of Origin recognition complex factors. Eur. J. Hum. Genet..

[73] Noguchi K., Vassilev A., Ghosh S., Yates J.L., DePamphilis M.L. (2006). The BAH domain facilitates the ability of human Orc1 protein to activate replication origins in vivo. EMBO J..

[74] Parker M.W., Bell M., Mir M., Kao J.A., Darzacq X., Botchan M.R. (2019). A new class of disordered elements controls DNA replication through initiator self-assembly. eLife.

[75] Adiji O.A., McConnell B.S., Parker M.W. (2024). The origin recognition complex requires chromatin tethering by a hypervariable intrinsically disordered region that is functionally conserved from sponge to man. Nucleic Acids Res..

[76] Hemerly A.S., Prasanth S.G., Siddiqui K., Stillman B. (2009). Orc1 controls centriole and centrosome copy number in human cells. Science.

[77] Gradia S.D., Ishida J.P., Tsai M.S., Jeans C., Tainer J.A., Fuss J.O. (2017). MacroBac: new technologies for robust and efficient large-scale production of recombinant multiprotein complexes. Methods Enzymol..

[78] Kiianitsa K., Solinger J.A., Heyer W.D. (2003). NADH-coupled microplate photometric assay for kinetic studies of ATP-hydrolyzing enzymes with low and high specific activities. Anal. Biochem..

[79] Zhang K. (2016). Gctf: real-time CTF determination and correction. J. Struct. Biol..

[80] Kimanius D., Dong L., Sharov G., Nakane T., Scheres S.H.W. (2021). New tools for automated cryo-EM single-particle analysis in RELION-4.0. Biochem. J..

[81] Mastronarde D.N. (2005). Automated electron microscope tomography using robust prediction of specimen movements. J. Struct. Biol..

[82] Punjani A., Rubinstein J.L., Fleet D.J., Brubaker M.A. (2017). cryoSPARC: algorithms for rapid unsupervised cryo-EM structure determination. Nat. Methods.

