# Supplementary material for: Molecular impacts of Meier–Gorlin syndrome mutations on human origin licensing[image]
Source: J Biol Chem. 2025 Dec 23;302(2):111100. doi: 10.1016/j.jbc.2025.111100 (PMC12830165; doi:10.1016/j.jbc.2025.111100)
Supplement: Supporting informtion [file mmc1.pdf]

Molecular impacts of Meier-Gorlin syndrome mutations on human origin licensing

Ran Yang, Olivia Hunker, Jenna Kim, and Franziska Bleichert*

Department of Molecular Biophysics and Biochemistry, Yale University, New Haven, CT, USA

* Correspondence: Franziska Bleichert
franziska.bleichert@yale.edu

Supporting Figures

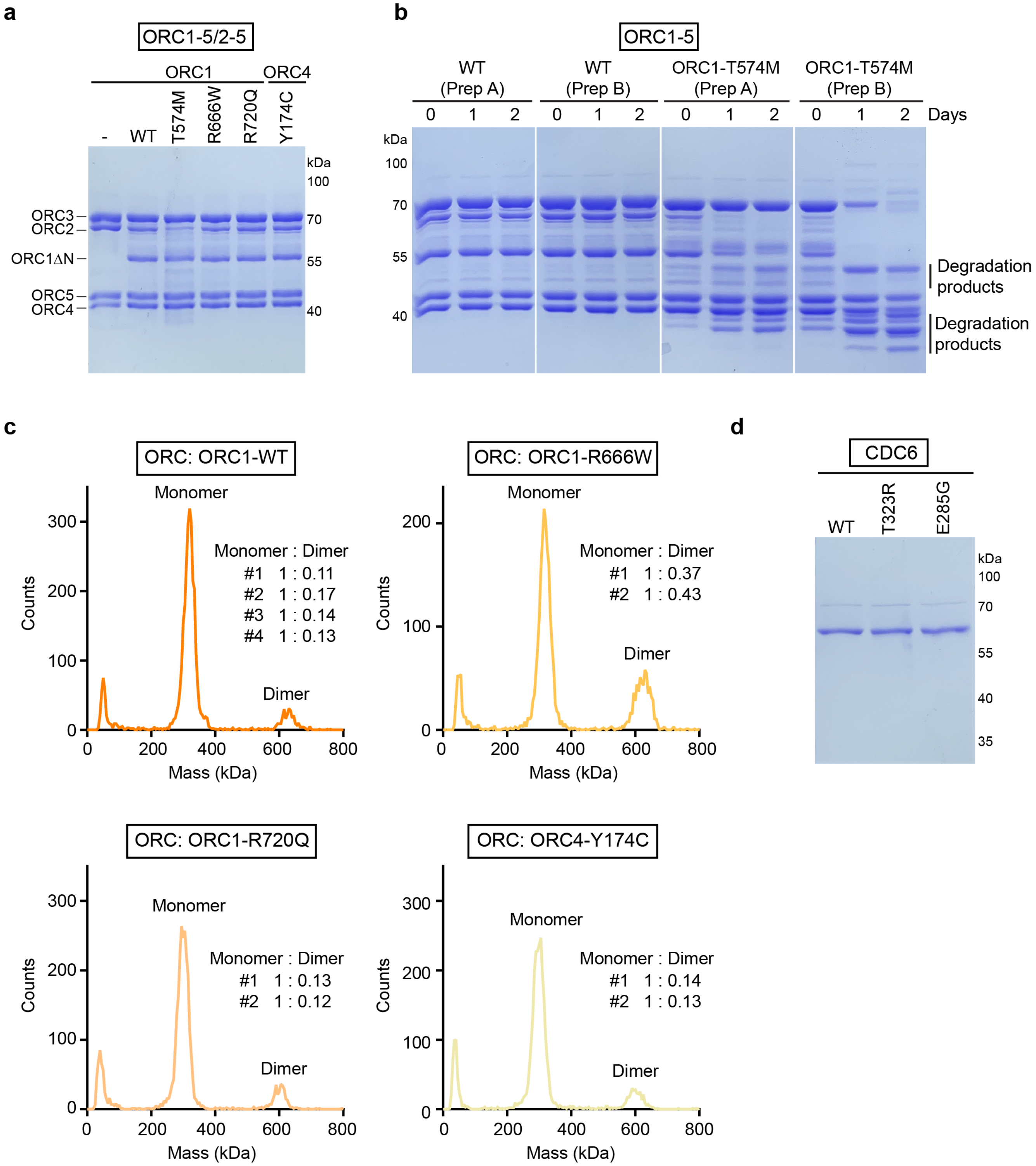

Supporting Figure S1. Purification and characterization of ORC and CDC6 MGS variants. **a**) Coomassie-stained SDS-PAGE gel of purified, recombinant human ORC assemblies, including wildtype or MGS-mutant ORC1-5 and ORC2-5 (without ORC1). **b**) The MGS mutation T574M in ORC1 destabilizes ORC, causing the degradation of several ORC subunits during and after protein purification. Coomassie-stained SDS-PAGE gel of two different purifications of wildtype ORC and ORC with ORC1^{T574M} stored on ice for the indicated time periods is shown. **c**) Mass photometry of wildtype

ORC and ORC containing MGS variants. ORC with ORC1^{R666W} shows an increase in ORC dimer formation. Monomer-to-dimer ratios for distinct repeat experiments are listed in each subpanel. **d**) Coomassie-stained SDS-PAGE gel of purified, recombinant human CDC6 proteins.

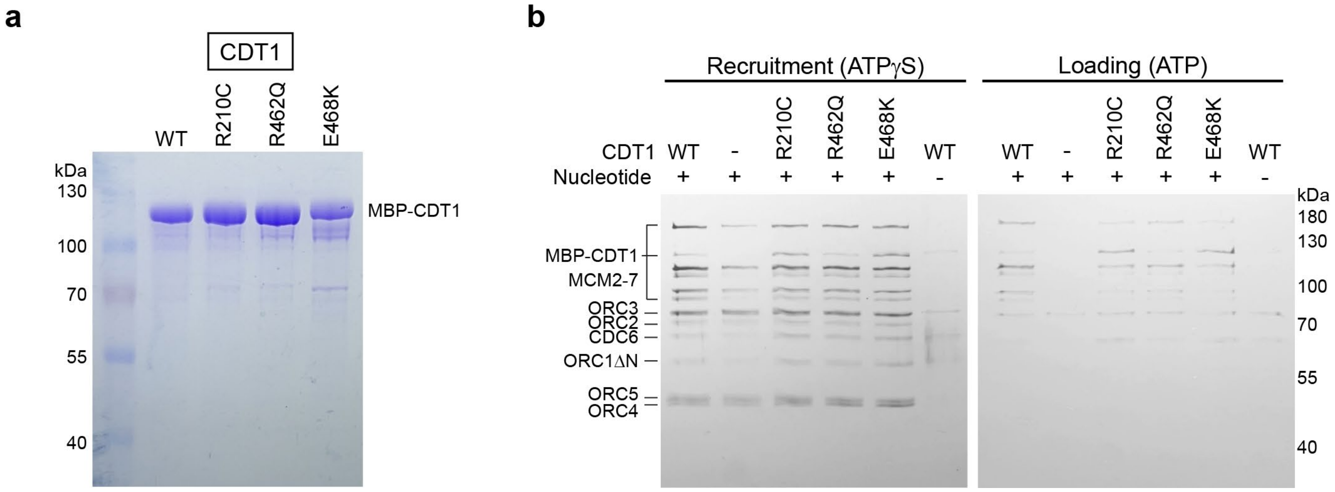

Supporting Figure S2. Purification and biochemical characterization of CDT1 variants. **a**) Coomassie-stained SDS-PAGE gel of purified, recombinant human CDT1 proteins (full-length). The MBP tag was retained to improve protein yields and stability. **b**) Silver-stained SDS-PAGE gel of elutions from MCM recruitment and loading assays. Note that full-length CDT1, especially R210C and E468K variants, binds non-specifically to beads.

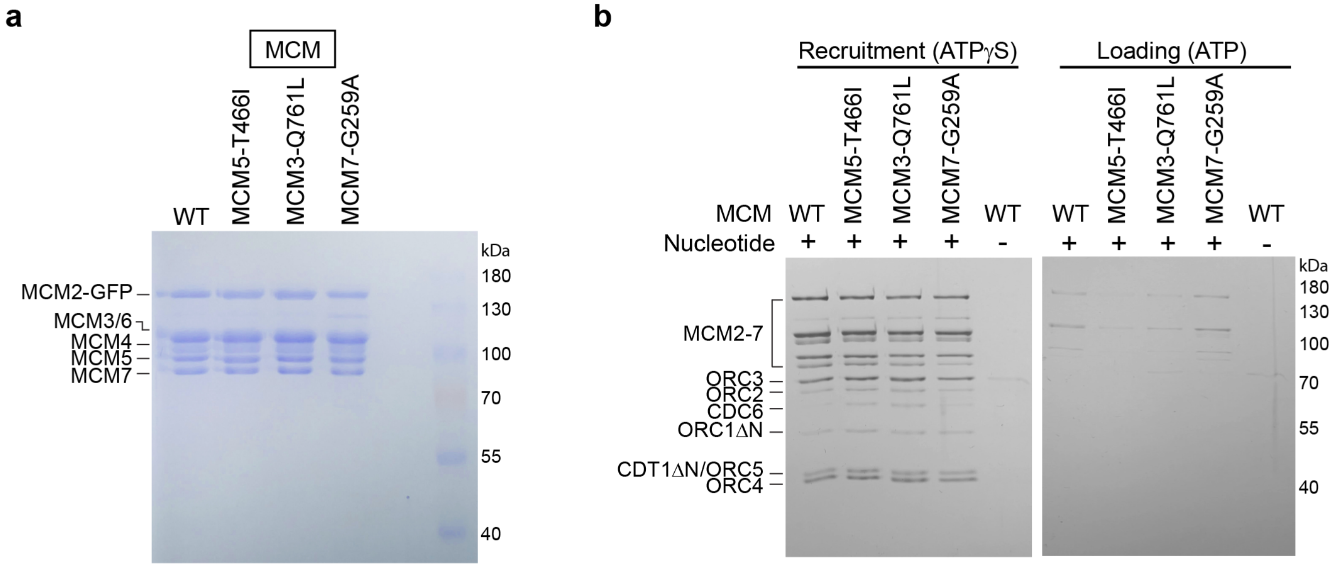

Supporting Figure S3. Purification and biochemical characterization of MGS-MCM assemblies. **a**) Coomassie-stained SDS-PAGE gel of purified, recombinant human MCM2-7 complexes. MCM2 contained a C-terminal GFP tag for fluorescence intensity measurements in MCM recruitment and loading assays. **b**) Silver-stained SDS-PAGE gel of elutions from MCM recruitment and loading reactions with wildtype or MGS-mutant MCM assemblies.
